# Recent advances in glycopeptide hydrogels: design, biological functions, and biomedical applications

**DOI:** 10.3389/fbioe.2025.1577192

**Published:** 2025-06-23

**Authors:** Jinpeng Zhang, Jianghao Zou, Jianan Ren

**Affiliations:** Research Institute of General Surgery, Jinling Hospital, School of Medicine, Nanjing University, Nanjing, China

**Keywords:** dynamic hydrogel, supramolecular hydrogel, self-assembling peptide hydrogel, injectable hydrogel, tissue engineering

## Abstract

Glycopeptide hydrogels, biomaterials constructed from polysaccharides and peptides through dynamic covalent bonding and supramolecular interactions, mimic the structure and functions of the natural extracellular matrix. Their three-dimensional network structure endows them with remarkable mechanical resilience, self-healing capacity, and stimuli-responsive behavior, enabling diverse biomedical applications in tissue regeneration, wound healing, drug delivery, and antimicrobial therapies. This review comprehensively examines design principles for engineering glycopeptide hydrogels, encompassing biomolecular selection criteria and dynamic crosslinking methodologies. We analyze their multifunctional properties including antimicrobial efficacy, immunomodulation, antioxidant activity, tissue adhesion, and angiogenic potential, while highlighting smart drug release mechanisms. Applications in regenerative medicine are critically assessed, particularly in cutaneous wound healing, bone and cartilage reconstruction, myocardial repair, and neural regeneration. Finally, we delineate future directions to advance glycopeptide hydrogels, emphasizing functional sequence expansion of bioactive motifs, high-fidelity biomechanical mimicry of natural tissues, and precise simulation of organ-specific microenvironments for next-generation precision medicine.

## 1 Introduction

Glycopeptide hydrogels, as an emerging class of multifunctional biomaterials, are typically constructed through the synergistic interplay of dynamic covalent bonds (e.g., Schiff base bonds, boronic ester bonds) and supramolecular interactions (e.g., hydrogen bonding, π–π stacking, electrostatic forces) between sugar molecules, peptide chains, or their functional analogs, forming a three-dimensional network structure. Compared to conventional hydrogels, glycopeptide hydrogels demonstrate superior dynamic responsiveness, enhanced bioactivity, and exceptional mechanical properties, offering innovative solutions to overcome the limitations of static cross-linked hydrogels in tissue repair and regeneration. Traditional hydrogels, constrained by static irreversible cross-linking, often suffer from insufficient mechanical strength, lack of self-healing capability, and poor adaptability to dynamic biological environments (e.g., pH fluctuations, oxidative stress). Additionally, their degradation rates are challenging to precisely control, and low loading efficiency of bioactive components further restricts their applications in chronic wound healing and tissue engineering. In contrast, glycopeptide hydrogels leverage dynamic covalent bonds to enable reversible network reorganization and self-healing properties, while supramolecular interactions enhance structural stability. Furthermore, the synergistic functionality of sugar and peptide moieties facilitates targeted drug delivery, cell adhesion, and behavior regulation. These advancements collectively achieve breakthroughs in smart responsiveness, biocompatibility, and therapeutic efficacy, positioning glycopeptide hydrogels as a transformative platform for advanced biomedical applications.

Sugar molecules are a core component of glycopeptide hydrogels, particularly natural polysaccharides such as hyaluronic acid (HA), chitosan, and alginates. These polysaccharides provide the necessary structural support for the hydrogel, have good biocompatibility and biodegradability, and exhibit a variety of inherent biological activities (Manzoor et al., 2022). For example, HA, by interacting with extracellular matrix receptors, can regulate the inflammatory microenvironment and promote cell adhesion and proliferation. Chitosan, through its cationic properties, exerts antimicrobial activity and promotes wound healing. Furthermore, conjugated monosaccharides and oligosaccharides such as D-mannose and D-glucose enhance the biocompatibility of the hydrogel and introduce specific targeting capabilities, further broadening the potential applications of hydrogels in precision medicine (Li J. et al., 2019; Teng et al., 2024; Teng et al., 2021).

Peptide molecules, with their high designability and diverse biological functions, such as cell adhesion, antimicrobial activity, and angiogenesis, have unique advantages in biomedical applications (Nazeer and Ahmed, 2025; Binaymotlagh et al., 2022). In addition, self-assembling peptides such as RADA16 (RADARADARADARADA) and diphenylalanine (FF)-derived peptides can form nanofiber networks that crosslink with polysaccharides to create a three-dimensional porous composite structure. This significantly enhances the material’s mechanical strength and better mimics the microstructure and dynamic properties of the natural extracellular matrix (ECM). The size of these nanofibers aligns with the scale of natural ECM fibers, and their dynamic crosslinking with polysaccharides further enhances their biochemical functionality and mechanical compatibility with native tissue, providing an ideal microenvironment for cell adhesion, proliferation, migration, and signal transmission (Guan et al., 2022; Castro et al., 2025; Zhao et al., 2024a).

Glycopeptide hydrogels are typically designed through dynamic crosslinking, using dynamic covalent bonds or non-covalent interactions to confer the material with self-healing properties, excellent viscoelasticity, and adaptability to complex environments. This dynamic characteristic effectively mimics the behavior of the natural ECM and enables the hydrogel to undergo controllable degradation and functional responses under external stimuli, such as changes in pH, temperature, and redox levels, thus enabling applications in complex biological environments (Zhang et al., 2021).

In recent years, glycopeptide hydrogels have demonstrated a dual trajectory of functional diversification and application precision. At the design level, researchers are actively exploring strategies such as multifunctional integration, multimodal microenvironmental responsiveness, biomimetic structural simulation, and immunomodulatory regulation to endow hydrogels with exceptional environmental adaptability and bioactivity. In practical applications, glycopeptide hydrogels have been widely utilized in neural injury repair, chronic refractory wound management (e.g., diabetic wounds, radiation-induced injuries), and bone and cartilage regeneration. These strategies emphasize the simulation of tissue-specific microenvironments, achieving multi-layered tissue regeneration and functional recovery by modulating immune responses, promoting angiogenesis, mitigating oxidative stress, and reconstructing extracellular matrices. Such innovations not only expand the functional boundaries of glycopeptide hydrogels but also lay a solid foundation for their clinical translation in precision medicine and regenerative medicine.

Although glycopeptide hydrogels demonstrate significant potential in biomedical applications, current research still faces several critical gaps. First, while classical RGD (Arg-Gly-Asp) sequences have been introduced to enhance cell adhesion, there remains a lack of systematic screening and functional reconstruction of other bioactive peptide motifs, limiting the material’s capacity for precise regulation in tissue-specific microenvironments. Second, although the mechanical properties of existing glycopeptide hydrogels—particularly their viscoelastic characteristics—have been improved via dual-network architectures or metal ion coordination, they still fall short of replicating the complex dynamic responsive behaviors of natural tissues such as skin. Third, the development of organ-specific bio-inspired ECM designs remains nascent, with insufficient systematic simulation of structure-function relationships across diverse organ microenvironments, thereby constraining the application of hydrogels in targeted tissue repair.

This review systematically outlines design principles and functional optimization strategies for glycopeptide hydrogels, elucidating their unique advantages in overcoming the limitations of conventional hydrogels and advancing tissue regeneration to accelerate clinical translation. A comprehensive analysis is presented across three critical dimensions: design strategies, biological functionalities, and practical applications. Building on current technical challenges, the review emphasizes future directions such as functional sequence expansion, high-fidelity biomechanical mimicry, and precise simulation of organ-specific microenvironments. A summary of reported glycopeptide hydrogel classifications (Table 1) is also included to provide researchers with a structured reference framework.

**TABLE 1:** Recent studies on glycopeptide hydrogels. The relationship between saccharides and peptides in hydrogels can be categorized as follows: monosaccharide-modified peptide chains, monosaccharide-modified self-assembling peptides, polysaccharides with peptide chains, and polysaccharides with self-assembling peptides. In addition, there are hydrogel systems composed of saccharide and peptide mimetics are included.

Mode	Saccharides	Peptides	Other components	Crosslinking method	Application	References
Monosaccharide-modified peptide chains	Glucose	PLL	FeCl_3_, horseradish peroxidase, and H_2_O_2_	Metal coordination crosslinking; phenolic oxidative coupling	Tissue adhesives and hemostatic materials	Teng et al. (2021)
Monosaccharide-modified self-assembling peptides	Glucosamine-6-sulfate	Fmoc- FF	Ca^2+^, Mg^2+^	Ionic crosslinking, peptide self-assembling	Cardiac tissue engineering and regeneration; Induction of stem cell differentiation into nerve cells	(Castro et al., 2025)/ (Castro et al., 2023)
	Maltopentaose	Nap-FF	-	Peptide self-assembling	Enzyme-activated prodrug therapy for tumors	Sakamoto et al. (2022)
	Galactose	Nap-FFSY (H_2_PO_3_)	-	Self-assembling triggered by alkaline phosphatase	Antibacterial materials	Liu et al. (2020)
	D-Mannose	Nap-FFSY (H_2_PO_3_)	-	Self-assembling triggered by alkaline phosphatase	Antibacterial materials	Li et al. (2019a)
	D-Glucosamine	Nap-FFDY (H_2_PO_3_)	Deferoxamine	Self-assembling triggered by alkaline phosphatase	Tissue regeneration (construction of vascular networks)	Qi et al. (2018)
	D-Glucosamine	Nap-FFDD	-	Peptide self-assembling	Investigation of glycosyl modification positions and quantities	Liu et al. (2016)
	Glucosamine	Fmoc-FFD	-	Peptide self-assembling	Scar inhibition after eye surgery	Xu et al. (2012)
Polysaccharides and peptide chains	Oxidized dextran, carboxymethyl chitosan	EPL	Extracellular vesicles	Schiff base	Meniscus repair	Huang et al. (2025)
	GM-CHO	ILPWKWPWWPWRR; DOPA_4_-G_4_-GRGDS; KSLSLSLRGSLSLSLKGKLTWQELYQLKYKGI	-	Schiff base, hydrogen bonding, hydrophobic interactions	Diabetic wound management	Liu et al. (2024)
	Oxidized chondroitin sulfate	KYKYEYEY	Aspirin	Schiff base	Radiation-induced skin injuries	Guo et al. (2024)
	Oxidized *Bletilla striata* polysaccharide	Gallic acid-grafted EPL	Amphiphilic micelles loaded with paeoniflorin	Schiff base	Chronic wound treatment	Zhang et al. (2024)
	Hydroxypropyl chitosan	EPL	2,3,4-Trihydroxybenzaldehyde, Fe^3+^	Schiff base, hydrogen bonding, metal coordination crosslinking	Wound healing	Peng et al. (2024)
	Oxidized dextran, gluconolactone	PLL	-	Schiff base	Cartilage repair	Hu et al. (2023)
	Oxidized alginate	Boronic acid-modified EPL	Epigallocatechin-3-gallate, salvianolic acid B@MnO_2_	Schiff base, boronate ester bond (pH/ROS dual response)	Chronic refractory wound dressings	Wu et al. (2023)
	Boronic acid-modified oxidized dextran	Caffeic acid-modified EPL	Diclofenac sodium, mangiferin-loaded micelles	Schiff base, boronate ester bond (pH/ROS dual response)	Chronic diabetic wound healing	Wu et al. (2022)
	GM-CHO, maleimide-modified HA	Antimicrobial peptide, collagen peptide (matrix metalloproteinase-responsive)	-	Schiff base, Michael addition	Chronic refractory skin wounds	Liu et al. (2022)
	Oxidized HA-glyceryl methacrylate	Thiolatedγ-PGA	-	Hemi-thioacetal bond, Michael addition	Wound repair	Yang et al. (2021a)
	Oxidized HA	Thiolated γ-PGA	-	Hemi-thioacetal reaction	Skin wound dressings	Yang et al. (2021b)
	Oxidized laminarin	Gelatin	-	Schiff base	ECM-mimetic hydrogel design	Lavrador et al. (2020)
	Oxidized salecan	Gelatin	Vancomycin	Schiff base	Drug delivery systems and tissue engineering	Fan et al. (2020)
	Oxidized dextran	EPL, partially acylated with acetic anhydride or succinic anhydride	-	Schiff base	Low-toxicity bioadhesive	Matsumura et al. (2014)
Polysaccharides and self-assembling peptides	GM-CHO	RADA16, W9 (WP9QY)	Minocycline hydrochloride	Schiff base, peptide self-assembling	Restoration of periodontal bone homeostasis	Zhao et al. (2024b)
	Chitosan	RADA16 and PPFLMLLKGSTR	-	Electrostatic and hydrogen bonding interactions, peptide self-assembling	Spinal cord injury repair	Sun et al. (2024)
	GM-CHO	R4K4 peptide (RADA16+KEYAKEYAKEYAKEY)	-	Schiff base	Wound management and tissue repair	Wang et al. (2024a)
	Fucoidan	NAP-FFGRGD	-	Hydrogen bonding and electrostatic interactions, peptide self-assembling	Cartilage repair and regeneration	Zhao et al. (2024a)
	Maleimide-modified GM	CK2(SL)6K2, decellularized ECM	-	Michael addition, peptide self-assembling	Cardiac repair post-myocardial infarction	Kong et al. (2023)
	Maleimide-modified GM	K2(SL)6K2	Tannic acid, metal ions	Michael addition, peptide self-assembling, metal coordination crosslinking	Radiation-induced skin injuries	Feng et al. (2023)
	GM-CHO	RADA16 peptide	Polycaprolactone and nano-hydroxyapatite as core scaffolds	Schiff base, peptide self-assembling	Cranial defect repair (immune modulation and osteoinduction)	Wang et al. (2022)
	GM-CHO	Q11 peptide (QQKFQFQFEQQ)	-	Schiff base, peptide self-assembling	Drug-free and cell-free wound healing	Feng et al. (2020)
	GM and alginate	Fmoc-FF	Docetaxel	Peptide self-assembling	Drug delivery	(Huang et al., 2011)/(Xie et al., 2016)
Saccharides and peptide mimetics	Polyvinyl alcohol	Boronic acid-modified EPL	Responsive micelles loaded with astragaloside IV	Boronate ester bond (pH/ROS dual response)	Chronic refractory wound dressings	Deng et al. (2024)
	D-Mannose, D-Glucose, polyethylene glycol N-hydroxysuccinimide-activated ester	Catechol-modified PLL	-	Ester-amine exchange reaction	Hemostatic dressings	Teng et al. (2024)
	Oxidized HA, oxime-modified HA	Dopamine-modified poly(6-aminohexanoic acid)	Sr^2+^	Schiff base, metal coordination crosslinking	pH-regulating diabetic chronic wound dressings	Xia et al. (2023)

EPL, ε-Poly-L-lysine; FF, diphenylalanine; Fmoc, Fluorenylmethoxycarbonyl; GM, glucomannan; GM-CHO, Oxidized glucomannan; Nap, Naphthalene; ROS, Reactive oxygen species; PLL, poly-L-lysine; RADA16, RADARADARADARADA; γ-PGA, γ-polyglutamic acid.

## 2 Saccharides commonly used in glycopeptide hydrogels

### 2.1 Polysaccharides

Polysaccharides, serving as the core components of glycopeptide hydrogels, play an indispensable role in material design due to their multifaceted biological functions and tunable chemical properties. The biological activities of polysaccharides are intricately linked to their chemical structures, particularly key parameters such as sugar unit types, functional group distribution, polymerization degree, and branching patterns. For example, sugar units like mannose and galactose form multivalent hydrogen-bonding networks with lectins (e.g., macrophage surface CD206 or bacterial surface LecA) through hydroxyl groups and cyclic structures, enabling specific molecular recognition and targeted interactions (Teng et al., 2024; Liu et al., 2020; Thalji et al., 2022). Furthermore, the glycocluster effect—enhanced by high-density arrangements of sugar units (e.g., β-1,4 glycosidic bonds in glucomannan)—significantly amplifies binding affinity, a critical feature for pathogen inhibition and immune modulation (Imberty and Varrot, 2008).

The hydroxyl, amino, and carboxyl functional groups in polysaccharides exhibit dual roles in chemical crosslinking and bioactivity. Hydroxyl groups can be oxidized to aldehyde (-CHO) groups to participate in dynamic Schiff base reactions. For instance, the dynamic covalent crosslinking between oxidized glucomannan (GM-CHO) and ε-polylysine (EPL) confers pH-responsive behavior to hydrogels (Matsumura et al., 2014). Simultaneously, hydroxyl groups scavenge free radicals via hydrogen atom transfer, as exemplified by Lycium barbarum polysaccharides (LBGP) neutralizing ROS to protect corneal epithelial cells (Wang Q. et al., 2024). The amino groups in chitosan disrupt bacterial membrane phospholipid bilayers through electrostatic interactions and activate the TLR4/MyD88 pathway to upregulate anti-inflammatory cytokines such as IL-10 (Geng et al., 2023). Carboxyl groups enable ionotropic crosslinking (e.g., the “egg-box” structures formed by alginate and Ca^2+^ (Wang et al., 2023)) or regulate pH-dependent drug release, such as protonation-triggered efficient delivery of paclitaxel in acidic microenvironments (Wang et al., 2018).

Polysaccharides can be classified into marine-, plant-, mammalian tissue-, and microbial-derived types based on their origins. The structural and functional properties of each polysaccharide determine its unique application potential in hydrogels. The following sections will explore the characteristics and applications of polysaccharides from these different sources in the design of glycopeptide hydrogels (Table 2).

**TABLE 2:** Comparison of polysaccharides in glycopeptide hydrogels: modification strategies and performance improvements.

Source	Polysaccharides	Main properties	Modification methods	Applications	Limitations
Marine - derived	Chitosan	Antibacterial, pH - sensitive, biodegradable, thermosensitive	Carboxymethylation, hydroxypropylation	Cartilage repair, wound healing, drug delivery, nerve repair	Poor solubility and low mechanical strength
	Fucoidan	Anti - inflammatory, immunomodulatory, antioxidant	-	Cartilage repair	High extraction cost and activity affected by the content of sulfate group
	Alginate	High biocompatibility, ionic crosslinking (e.g., Ca^2+^), pH - responsive, antibacterial	Oxidation	Wound repair, tissue engineering, drug delivery	Low mechanical strength
	Laminarin	Antioxidant, anti - inflammatory, immunomodulatory, high fluidity	Oxidation	Tissue engineering	Poor structural stability, easy to be hydrolyzed by enzyme
Plant - derived	Glucomannan	Anti - inflammatory, immunomodulatory	Oxidation, modification with maleimide groups	Tissue repair	Insufficient swelling capacity and poor control release characteristics
	*Bletilla striata* polysaccharide	Antioxidant, antibacterial, anti - aging, promotes wound healing	Oxidation	Wound dressing, skin repair	Complex extraction process, high industrial production cost
Mammalian tissue - derived	Hyaluronic acid	High hydrophilicity, biocompatibility, promoting cell migration and proliferation	Oxidation, methacrylation, modification with maleimide groups	Wound dressing, cartilage repair	Easily degraded by hyaluronidase *in vivo*, low mechanical strength
	Chondroitin sulfate	High negative charge, anti - inflammatory, promoting cell adhesion and migration	Oxidation	Cartilage repair	Limited source (animal extraction), difficult purification
Microbial - derived	Dextran	High water solubility, chemically modifiable, good biocompatibility	Oxidation	Cartilage repair, tissue adhesives	May cause immune response, needs to control the molecular weight distribution
	Salecan	Immune - modulating, biodegradable, excellent biocompatibility	Oxidation	Controlled drug release, tissue repair	Few studies, long-term biosafety needs to be verified

#### 2.1.1 Marine-derived polysaccharides

Chitosan, a cationic polysaccharide derived from chitin, is composed of N-acetylglucosamine and glucosamine units. It is the second most abundant natural polysaccharide after cellulose and has garnered attention in the design of glycopeptide hydrogels due to its excellent polycationic properties, antibacterial activity, and bioabsorbable nature (Shariatinia and Jalali, 2018). Chitosan interacts with negatively charged microbial cell surfaces and intracellular components, exerting antibacterial effects by altering membrane permeability (MubarakAli et al., 2018). Chitosan is pH-sensitive, as it is soluble at a low pH and insoluble at a high pH, rendering it highly applicable for controlled release systems (Treenate and Monvisade, 2017). Modifications, such as carboxymethylation (Huang et al., 2025) and hydroxypropylation (Peng et al., 2024; Baranwal et al., 2018), significantly enhance its water solubility, while incorporating thermosensitive molecules (e.g., β-glycerophosphate) (Wang X. et al., 2024) imparts the hydrogel with thermosensitive properties and improved controlled release capabilities. These modifications have greatly expanded the use of chitosan in applications such as wound healing, tissue engineering, and drug delivery.

Fucoidan, a sulfated polysaccharide derived from brown algae and characterized by its sulfate-rich structure, has important biological functions, such as antioxidant, anti-inflammatory, and immunomodulatory activities (Marinval et al., 2018; Zhu et al., 2021; Yao et al., 2020). Due to its structural similarity to chondroitin sulfate, fucoidan has tremendous potential in cartilage repair and regeneration (Zhao et al., 2024a).

Alginate, a water-soluble linear polysaccharide extracted from brown algae, is composed of alternating β-D-mannuronic acid and α-L-guluronic acid residues. It exhibits excellent biocompatibility, biodegradability, and antibacterial properties, promotes wound healing, and functions as a drug carrier, making it widely used in wound healing, tissue repair (Varaprasad et al., 2020; Del Gaudio et al., 2020; Moura et al., 2013), and drug delivery systems (George and Abraham, 2006). When combined with metal cations (e.g., Ca^2+^), alginate undergoes ionic crosslinking to form hydrogel networks, enhancing its drug encapsulation and pH-responsive properties and thus serving as a responsive controlled-release platform (Xie et al., 2016; Treenate and Monvisade, 2017).

Laminarin is a low-molecular-weight β-glucan derived from brown algae consisting of (1,3) and (1,6) glycosidic bonds in varying proportions (O’Sullivan et al., 2010). It exhibits antioxidant, antitumor, immunomodulatory, and wound-healing effects, which contribute to disease prevention (Sellimi et al., 2018; Tümen ERDEN-Ceyda Ekentok, 2021; Miao et al., 1999). Compared with other natural polysaccharides (e.g., alginate and chitosan), the lower molecular weight of laminarin has superior flowability and processability during hydrogel preparation (Lavrador et al., 2020).

#### 2.1.2 Plant-derived polysaccharides

Glucomannan (GM), a natural polysaccharide comprising mannose and glucose units linked by β-1,4-glycosidic bonds, is one of the most common polysaccharides in glycopeptide hydrogels. GM is rich in mannose residues, which specifically bind to mannose receptors on macrophage surfaces. This property has been widely exploited to promote macrophage polarization to the anti-inflammatory M2 phenotype, indirectly enhance fibroblast proliferation and angiogenesis, and accelerate tissue repair and regeneration (Liu et al., 2022; Zhao et al., 2024b; Wang J. et al., 2024). In glycopeptide hydrogels, oxidizing GM introduces dynamic crosslinking with peptides (Liu et al., 2024; Wang et al., 2022; Feng et al., 2020). By adding maleimide groups, GM can specifically react with thiol groups on peptides, further enhancing its peptide crosslinking selectivity (Kong et al., 2023; Feng et al., 2023).

*Bletilla striata* polysaccharide (BSP) is a natural glucomannan extracted from the traditional Chinese medicinal herb *B. striata*. It consists of mannose (α-mannose and β-mannose) and β-glucose at a molar ratio of 3:1 and 2.4:1. The key biological functions of BSP include wound healing, antioxidative and antibacterial effects, and anti-aging activity (Bai et al., 2024). BSP-based wound dressings have shown strong potential for applications in wound healing and repair (Gou et al., 2022; Xu et al., 2023; Luo et al., 2010).

#### 2.1.3 Mammalian tissue-derived polysaccharides

HA is a naturally occurring glycosaminoglycan composed of repeating disaccharide units of D-glucuronic acid and N-acetyl-D-glucosamine. It is widely distributed in the extracellular matrix, synovial fluid, and skin of mammals (Bhattacharya et al., 2017). The unique rheological properties of HA derive from its molecular backbone and variable secondary and tertiary structures, enabling it to adapt to diverse physical and biological environments. HA exhibits high hydrophilicity, excellent biocompatibility, and biodegradability, and is essential for maintaining tissue homeostasis and promoting cell adhesion, migration, and proliferation (Wu et al., 2024). Because of these properties, HA is extensively used in biomedical applications (Dicker et al., 2014). However, natural HA also has limitations, such as poor stability, sensitivity to hyaluronidase and free radicals, a short half-life *in vivo*, and insufficient mechanical strength in aqueous systems (Lee et al., 2018). To resolve these issues, the HA structure and properties are typically modified. In glycopeptide hydrogels, introducing chemical modifications, such as methacrylate or maleimide groups, enables HA to dynamically couple with thiol-containing or stimulus-responsive peptide molecules (e.g., collagen tripeptides responsive to matrix metalloproteinases) (Liu et al., 2022; Yang et al., 2021a). These modifications then facilitate the dynamic degradation and functionalization of hydrogels.

Chondroitin sulfate, also a sulfated glycosaminoglycan, consists of repeating disaccharide units of D-glucuronic acid and N-acetyl-D-galactosamine. It is commonly found in cartilage, connective tissue, and the ECM. Unlike HA, chondroitin sulfate is often sulfated at hydroxyl groups in the four or six position, which imparts a highly negative charge. Chondroitin sulfate participates in tissue repair by promoting cell adhesion and migration, modulating inflammatory responses, and regulating cellular behaviors through signaling molecule interactions (Faheem et al., 2024).

#### 2.1.4 Microbial-derived polysaccharides

Dextran is an extracellular polysaccharide derived from microorganisms that comprise a linear backbone of glucose molecules linked by α-1,6-glycosidic bonds, with branches formed by α-1,3, α-1,4, or α-1,2 linkages. It is synthesized mainly by lactic acid bacteria and other microorganisms and is known for its high biocompatibility and low toxicity (Díaz-Montes, 2021). Dextran has excellent water solubility; tunable branch positions, molecular lengths, and molecular weights; and abundant chemical modification sites, such as hydroxyl groups, and therefore is widely used in drug delivery, wound healing, and tissue engineering (Zhao and Jalili, 2022; Rebello and Mazumder, 2024; Pacelli et al., 2021). Dextran can be oxidized or functionalized with boronic acid groups to form dynamic hydrogels with responsive properties for smart drug delivery systems (Wu et al., 2022).

Salecan is a newly discovered bacterial polysaccharide from the halophilic strain *Agrobacterium* ZX09, which consists of D-glucose units connected by α-(1–3) and β-(1–3) glycosidic bonds (Xiu et al., 2010). Salecan has excellent immunostimulatory, biocompatible, and biodegradable properties (Qi et al., 2019). Salecan-based hydrogels are applied in controlled drug release, three-dimensional (3D) cell culture, and tissue repair.

### 2.2 Monosaccharides and oligosaccharides

While polysaccharides often serve as the matrix or backbone in glycopeptide hydrogels, monosaccharides and oligosaccharides are commonly used as peptide modifiers to improve the biocompatibility and biophysical properties of hydrogels (Liu et al., 2016). Monosaccharides and oligosaccharides provide specific antibacterial targeting, glycosaminoglycan mimicry, and other biological functions required by various biomedical applications. Monosaccharide-based modifications are typically designed according to specific application requirements using a modular approach to provide diverse biological functionality.

#### 2.2.1 Monosaccharides and their derivatives

Monosaccharides and their derivatives (e.g., D-mannose, D-glucosamine, D-glucose, galactose) are widely used to chemically modify peptide side chains due to their simple structures and abundant reactive sites.

For example, D-mannose has been used for specific O-mannosylation of peptide chains that enhance the binding affinity of the hydrogel to target proteins, such as adhesins on bacterial surfaces and mannose receptor-expressing macrophages. This binding facilitates antibacterial, immunomodulatory, and pathogen-targeting activities. Specifically, the mannose modifications mimic the multivalent binding systems of natural sugar-lectin interactions, which induce bacterial adhesion, aggregation, and membrane disruption to selectively kill bacteria (Li J. et al., 2019). Mannose also interacts with macrophages to promote anti-inflammatory M2 macrophage polarization, which reduces inflammatory responses and suppresses the expression of pro-inflammatory cytokines, such as tumor necrosis factor-α (TNF-α) (Teng et al., 2024).

Similarly, glycopeptide hydrogels modified with galactose exploit the multivalent interactions between galactose and lectins to specifically target bacteria, inhibiting biofilm formation and bacterial growth (Liu et al., 2020).

D-glucose is typically covalently linked via its hydroxyl (–OH) group to the peptides of a hydrogel, mimicking natural glycosylation structures. This provides localized metabolic support to cells cell proliferation and tissue regeneration. In addition, this modification can indirectly increase biocompatibility by enhancing the hydrogel hydrophilicity and reducing the hemolytic rate (Teng et al., 2021).

D-glucosamine-modified peptides mimic core protein proteoglycans, enhancing the hydrogel hydrophilicity and biocompatibility while strengthening cell–hydrogel interactions and modulating cellular behavior. For example, these modifications inhibited the activity of transforming growth factor-β (TGF-β) to reduce fibroblast over proliferation and postoperative fibrosis (Xu et al., 2012). They were also shown to promote cell adhesion and proliferation through sugar–receptor interactions (Qi et al., 2018; Liu et al., 2016).

Glucosamine-6-sulfate-modified short peptides mimic the biological actions of sulfated proteoglycans, such as the induction of stem cell differentiation (Castro et al., 2023). In addition, these modifications enhance the ability of hydrogels to propagate electrical signals, making them suitable for cardiac tissue engineering and regeneration (Castro et al., 2025).

#### 2.2.2 Oligosaccharides

Oligosaccharides, with their relatively high molecular complexity, form rich hydrogen-bonded networks through interactions among multiple hydroxyl groups. Maltopentaose, an oligosaccharide composed of five glucose units, exhibits a high hydration capacity and excellent biocompatibility. When covalently bound to an FF backbone, maltopentaose-modified amphiphilic glycopeptides provided a hydrated environment that maintained enzymatic biocatalytic activity, rendering the hydrogels suitable for enzyme-activated prodrug therapies. In addition, maltopentaose endowed the hydrogels with remarkable shear-thinning properties and good injectability (Sakamoto et al., 2022).

## 3 Peptide design for glycopeptide hydrogels

The design of peptides for use in glycopeptide hydrogels is driven by three main functions: self-assembling and structural support, bioactive functionality, and dynamic regulation and signal responsiveness. By selecting appropriate peptide sequences and modification approaches, different types of peptides confer the hydrogel matrix with specific functional attributes.

### 3.1 ε-Poly-L-lysine (EPL)

EPL is a natural cationic antimicrobial peptide produced by *Streptomyces albulus*. Unlike other antimicrobial peptides (AMPs) that are often hemolytic or toxic, EPL is edible, non-toxic, biodegradable, and highly biocompatible. It is resistant to thermal degradation and can be produced at a large scale cost-effectively by fermentation (Shima et al., 1984; Hyldgaard et al., 2014; Wang et al., 2016). EPL has been approved by the Food and Drug Administration, is widely applied in clinical practice, and is one of the most common peptides used for developing glycopeptide hydrogels. EPL exerts potent antibacterial effects by disrupting bacterial cell membranes through electrostatic interactions and therefore has a low likelihood of inducing bacterial resistance (Yan Y. et al., 2021). Moreover, its cationic nature induces a series of hemostatic responses, including platelet and erythrocyte aggregation (Peng et al., 2024). EPL contains abundant lysine residues, which can replace hydrated cations on wet tissue surfaces and provide additional amino groups, thereby enhancing the adhesion of wet materials (Zhu et al., 2023). Its degradation product, lysine, also promotes tissue repair and regeneration (Huang et al., 2025).

EPL can be chemically functionalized through various modification strategies. For example, incorporating gallic acid (GA) or epigallocatechin-3-gallate (EGCG) enhanced its antioxidative properties (Zhang et al., 2024; Wu et al., 2023). In addition, dual-dynamic crosslinking with phenylboronic acid or 2,3,4-trihydroxybenzaldehyde imparted hydrogels with stimuli-responsiveness and significantly improved their mechanical performance and environmental adaptability (Peng et al., 2024; Wu et al., 2023).

### 3.2 Poly-L-lysine (PLL)

PLL is a polypeptide chain formed by linking lysine molecules through their α-amino and carboxyl groups. Unlike EPL, the high charge density of PLL confers it with some cytotoxicity; thus, it is often modified with mannose or glucose to improve its biocompatibility (Teng et al., 2024; Hu et al., 2023). By modifying PLL with catechol, stable covalent bonds can be formed with amino and thiol groups on tissue surfaces, which significantly enhances its adhesion in moist tissue environments. In addition, this modification can use metal ion coordination for dual-dynamic crosslinking or use quinone groups to further introduce covalent crosslinking (Teng et al., 2021). Crosslinking PLL with polyethylene glycol N-hydroxysuccinimide-activated ester (PEG-NHS) further optimizes the mechanical properties of the hydrogel. Its rapid shaping ability and the large pore structures significantly accelerate the hemostatic process and tissue repair (Teng et al., 2024).

### 3.3 Poly-γ-glutamic acid (γ-PGA)

γ-PGA is a natural polypeptide produced through microbial fermentation. Due to its excellent biocompatibility and biodegradability, it is used to produce glycopeptide hydrogels. Yang and colleagues (Yang et al., 2021b) modified γ-PGA with L-cysteine to generate thiolated polyglutamic acid (γ-PGA-SH), which endowed the material with excellent antioxidant properties. In addition, by introducing thiol groups, sites were provided for dynamic crosslinking with polysaccharides, which promoted covalent bonds to form through maleimide addition reactions, thus modulating the stiffness of the hydrogel (Yang et al., 2021a).

### 3.4 Gelatin

Gelatin is a natural protein derived from the hydrolysis of collagen. It consists of three polypeptide chains that form a triple helix structure through intermolecular hydrogen bonds. Gelatin has a number of useful biological properties, including high biocompatibility, high biodegradability, and rich functional sequences, including RGD sequences and matrix metalloproteinase-sensitive sequences. These specific functional domains mimic natural peptide functions in the ECM. In addition, gelatin has abundant side chains that are easily modified, and therefore it is widely used in biomedical hydrogels (Jaipan et al., 2017).

### 3.5 Self-assembling peptides

#### 3.5.1 RADA16 peptide

RADA16 is a typical type I ion-complementary self-assembling peptide in the self-assembling peptide family. It self-assembles into a β-sheet superstructure through hydrogen bonding and hydrophobic effects. In an acidic aqueous solution and when exposed to fluids at a physiological pH, RADA16 rapidly self-assembles into a network hydrogel structure within seconds (Zhao et al., 2008; Yokoi et al., 2005). The resulting structure mimics the fibrous morphology of the natural ECM and provides a favorable environment for cell adhesion and proliferation (Wang et al., 2017). RADA16 is an excellent carrier for delivering cells, drugs, and specific factors and therefore is useful in controlled molecular release applications (Nagai et al., 2006; Yao et al., 2023). However, RADA16 is limited by its weak mechanical properties and poor hydrophilicity. In glycopeptide hydrogels, RADA16 has been co-assembled with polysaccharides such as chitosan (Sun et al., 2024), oxidized GM (Zhao et al., 2024b; Wang J. et al., 2024; Wang et al., 2022), and functional short peptides such as WP9QY (W9) (Zhao et al., 2024b) and PPFLMLLKGSTR (Sun et al., 2024) to form composite hybrid hydrogels that improved the RADA16 hydrogel hydrophilicity and mechanical strength while imparting it with new biological activity. In addition, RADA16 can act as a self-assembling group coupled with functional sequences. In a study by Wang et al. (Wang J. et al., 2024), RADA16 was chemically conjugated to the T-cell epitope KEYA16 to form R4K4, while retaining its ability to self-assemble, it also induced antigen-specific type 2 immune responses.

#### 3.5.2 Phenylalanine (FF)-derived peptides

The FF motif is a self-assembling structural unit that exhibits strong supramolecular interactions in aqueous environments and easily forms β-sheet structures (Azuri et al., 2014). By combining aromatic groups such as Nap or Fmoc groups with the FF motif, hydrophobicity and π–π stacking are further enhanced, and the peptide is driven to form stable nanofiber networks. When co-crosslinked with polysaccharides, such as in glycopeptide hybrid hydrogels, the synergistic non-covalent and ionic interactions significantly improve the stability and mechanical strength of the hydrogels, thereby expanding their range of applications (Huang et al., 2011; Xie et al., 2016). Furthermore, by introducing responsive or functional modifications such as glycosylation, phosphorylation, or RGD sequences into FF-based self-assembling motifs, both the hydrogel stability and biological activity are synergistically enhanced. For example, Sakamoto et al. (Sakamoto et al., 2022) linked hydrophilic maltopentaose to the Nap-FF group and prepared injectable biocatalytic supramolecular hydrogels. The hydrogel was loaded with enzymes based on an amphiphilic glycopeptide design using a simple enzyme solution and has potential uses in enzyme-mediated prodrug therapy. Modifications using monosaccharides are more common, with glucose (Qi et al., 2018; Liu et al., 2016; Xu et al., 2012), mannose (Li J. et al., 2019), and galactose (Liu et al., 2020) linked to an Asp side chain via amide bonds. During the self-assembling process, the sugar moieties are exposed on the nanofiber surface, forming multivalent sugar cluster structures that perform the biological functions of the sugar groups while also increasing the hydrogel solubility, stability (resistance to proteolytic degradation), and biocompatibility. Fmoc-FF chemically conjugated to glucosamine-6-sulfate (GlcN6S) represents a minimal mimicry of sulfated proteoglycans (Castro et al., 2025; Castro et al., 2023). Another typical design involves introducing phosphorylated tyrosine (Tyr(H_2_PO_3_)), in which dephosphorylation is triggered under the action of alkaline phosphatase, significantly increasing the molecule’s hydrophobicity and inducing the peptide to self-assemble into nanofibers (Li J. et al., 2019; Liu et al., 2020; Qi et al., 2018). Zhao et al. (Zhao et al., 2024a) designed a Nap-FFGRGD peptide, which used the RGD sequence for integrin binding to create a favorable microenvironment for *in situ* stem cells with hydrogels, exhibiting both cell adhesion and signal transduction functionality. Other sequence designs have also been generated based on specific biological needs. For example, modifying Nap-FFDD with hydrophilic residues improved the thermal stability and biocompatibility of the assembled structure, generating a stable cell scaffold material (Liu et al., 2016). Similarly, an FMOC-FFD design that incorporated Asp improved the solubility and overall performance of the assembly (Xu et al., 2012).

#### 3.5.3 Q11 peptide

The Q11 peptide (QQKFQFQFEQQ) is a β-sheet peptide that self-assembles into highly ordered, β-sheet-rich nanofiber structures in a saline environment (Jung et al., 2009), triggering hydrogels to form Feng et al. (2020).

### 3.6 Functional short peptides

#### 3.6.1 Antioxidant peptides

The antioxidant activity of the KYKYEYEY peptide relies on the phenolic groups in its tyrosine residues, which interact with superoxide anions (O_2_^−^·) and other free radicals through hydrogen atom donation to effectively reduce oxidative stress and accelerate tissue repair (Hao et al., 2023). Its lysine residues also participate in chemical crosslinking reactions, enhancing the structural stability of the hydrogel (Guo et al., 2024). The DOPA_4_-G_4_-GRGDS peptide derived from mussels contains dopamine groups and an RGD sequence. The DOPA group directly scavenges superoxide anions and hydroxyl radicals through redox reactions, which improves the microenvironment of chronic wounds, while the RGD sequence enhances cell adhesion and proliferation (Pan et al., 2016).

#### 3.6.2 Angiogenesis peptides

The KK peptide (KKSLSLSLSLSLSLKK) is a self-assembling amphiphilic peptide with a β-sheet secondary structure, high solubility, and supramolecular assembly in aqueous solutions (Galler et al., 2010). Notably, hydrogels with KK peptides provide a high degree of cellular infiltration *in vivo*, thus inducing angiogenesis and attracting neural innervation without the need to deliver exogenous bioactive components (Moore et al., 2018). The PAP peptide (K(SL)3RG(SL)3KGKLTWQELYQLKYKGI) is an injectable self-assembling biodegradable nanofiber scaffold. Based on the KK peptide sequence, it incorporates a peptide mimic derived from VEGF-165 (vascular endothelial growth factor), thereby enhancing angiogenesis (Kumar et al., 2015).

#### 3.6.3 AMPs

AMPs are short peptides produced by many organisms to inhibit microbial pathogen proliferation. Although AMPs exhibit strong antimicrobial activity, their high toxicity to microorganisms and mammalian cells, along with their low selectivity, limits their clinical applications (Sun et al., 2018). In glycopeptide hydrogels, AMPs such as ILPWKWPWWPWRR have been combined with natural polysaccharides, such as GM-CHO, to simultaneously maintain antimicrobial activity while reducing toxicity (Liu et al., 2024; Liu et al., 2022).

#### 3.6.4 Other functional peptides

The W9 peptide mimics osteoprotegerin, which inhibits osteoclast maturation by blocking the RANKL/RANK signaling pathway, and thus promotes bone regeneration and prevents bone resorption (Aoki et al., 2006). The PPFLMLLKGSTR peptide is a motif derived from the laminin-5 α3 chain, which was shown to be the primary binding site for α3β1 integrin (Kim et al., 2005). This peptide strongly promotes stem cell adhesion and neuro tissue bridging and has been used for treating nerve injuries and neurodegenerative diseases (Li L. et al., 2019). Collagen peptide, an enzymatic hydrolysis product of collagen, contains sequences such as Gly-Pro-Hyp and supports cell adhesion to promote cell proliferation and migration, thus accelerating wound healing (Fahmy‐Garcia et al., 2018).

## 4 Dynamic crosslinking strategies

Dynamic crosslinking strategies have become a core design concept in the development of highly functional biomimetic glycopeptide hydrogels. Through dynamic crosslinking, hydrogels can mimic key characteristics of natural extracellular matrices, including their viscoelasticity, adaptability, and biological complexity (Chaudhuri et al., 2016; Chaudhuri et al., 2020). By contrast, the rigid structures of traditional hydrogel networks are difficult for cells to remodel, which hinders cell proliferation and migration and limits their use in regenerative medicine (Rosales and Anseth, 2016; Wang and Heilshorn, 2015).

Dynamic crosslinking constructs dynamic networks through reversible chemical bonds and non-covalent interactions, granting hydrogels excellent injectability, tissue adhesion, and responsiveness to pH or ROS changes (Zhang et al., 2021). Dynamic crosslinking includes dynamic covalent bonds, such as Schiff base, boronate ester, and hemithioacetal bonds, as well as non-covalent interactions, such as hydrogen bonds, hydrophobic interactions, electrostatic interactions, and metal coordination. Dynamic covalent bonds exhibit reversibility, environmental responsiveness, and high chemical stability, and therefore regulate the crosslinking density, mechanical strength, and degradation rate of hydrogels. Non-covalent interactions, the core of a supramolecular strategy, impart excellent self-healing and stimulus-responsive capabilities to hydrogels through weak and dynamic interactions (Li et al., 2024). In particular, supramolecular designs further improve the interface adhesion between hydrogels and biological tissues through a complex hierarchical and dynamic microenvironment that enhances biomimetic properties (Omar et al., 2022; Zhao et al., 2022). By combining dynamic covalent bonds with supramolecular strategies, glycopeptide hydrogels more efficiently dissipate energy, adapt to extreme environments, and achieve precise functional regulation. Table 3 summarizes the common dynamic crosslinking methods used in glycopeptide hydrogels.

**TABLE 3:** Common dynamic crosslinking methods used in glycopeptide hydrogels.

Crosslinking method	Main mechanism	Dynamic characteristic	Strength	Weakness
Schiff base	Aldehyde/ketone groups react with amino/hydrazide groups to form imine bonds (C=N)	pH-dependent reversibility; stable at neutral/alkaline pH, hydrolyzes in acidic conditions	Rapid gelation; biocompatible degradation; tunable crosslinking density	Limited stability at physiological pH (≈7.4); cytotoxic risks from excess aldehyde groups
Boronate ester bond	Boric acid/derivatives react with cis-diols to form cyclic boronate esters	pH/ROS/glucose-responsive; reversible switching between sp^2^ (neutral) and sp^3^ (ionized) forms	ROS scavenging; glucose sensing; self-healing; injectable	Instability at physiological pH; slow kinetics in neutral pH; competitive sugar interference
Hemithioacetal bond	Thiol-aldehyde addition forms R−CH(SR')−OH structures	Rapid exchange reactions; low pH dependence; reversible under physiological conditions	Fast gelation; biothiol-triggered dissolution; mild processing	Oxidation-sensitive (forms disulfides); moderate mechanical strength; requires thiol protection
Metal coordination crosslinking	Transition metal ions (Fe^3+^, Zn^2+^) coordinate with ligands (e.g., polyphenols)	Tunable kinetics via metal/ligand design; reversible “on-off” switching via chelators	High stiffness-ductility synergy; self-healing; conductivity modulation	Metal ion cytotoxicity risks; pH/temperature sensitivity; network instability in complex biofluids
Weak non-covalent interactions	Hydrogen bonds, π–π stacking, electrostatic interactions	Rapid self-recovery; stimulus-responsive (pH, temperature, ionic strength)	Extreme deformability; error-correction capability; adaptable mechanics	Low intrinsic strength; instability under high ionic strength or competing molecules

Compared with traditional hydrogels, glycopeptide hydrogels based on dynamic crosslinking demonstrate superior mechanical properties and functional diversity. These hydrogel designs expand the application potential of glycopeptide hydrogels in the biomedical field and provide ample opportunity for developing high-performance materials that are self-healing, degradable, and display intelligent responsiveness.

### 4.1 Schiff base

Schiff base bonds are a pivotal crosslinking mechanism in dynamic covalent hydrogels, formed through reversible reactions between aldehyde/ketone groups and amino (or hydrazide) groups. The reaction mechanism involves three key steps: (i) Nucleophilic addition, where the nucleophilic nitrogen atom of amino (-NH_2_) or hydrazide (-NH-NH_2_) groups attacks the carbonyl carbon of aldehyde (-CHO) or ketone (C=O) groups, forming a dipolar tetrahedral intermediate; (ii) Proton transfer and dehydration, characterized by intramolecular proton transfer from nitrogen to oxygen to generate amino alcohol, followed by acid-catalyzed dehydration to yield iminium ions; and (iii) Deprotonation, where iminium ions lose protons to form stable Schiff base bonds (C=N) while releasing catalytic hydronium ions (H_3_O^+^) to complete the cycle (Sahajpal et al., 2022).

The dynamic reversibility of Schiff base bonds stems from their pH sensitivity, maintaining stability under physiologically neutral or alkaline conditions (pH 7.0–8.0) but undergoing protonation and hydrolysis into original aldehyde and amino groups in acidic microenvironments (pH < 6.5, e.g., tumor tissues or chronic wounds) (Mo et al., 2021). This pH-responsive characteristic makes them particularly suitable for designing intelligent hydrogels (Wu et al., 2022). Notably, aldehyde groups (e.g., -CHO from oxidized glucomannan) exhibit superior reactivity in Schiff base formation compared to ketones due to reduced steric hindrance, enabling their widespread application in hydrogel design. In glycopeptide hydrogels, dynamic networks formed through aldehyde-amine interactions confer exceptional self-healing properties and environmental responsiveness. Natural polysaccharides can be readily crosslinked with peptides through oxidation-generated aldehyde groups. Precise control over hydrogel crosslinking density, mechanical strength, and degradation rate is achievable by regulating aldehyde group density through adjustment of NaIO_4_ oxidant molar ratios, facilitating tailored functional applications (Lavrador et al., 2020). Importantly, the aldehyde-to-amine ratio not only influences gel formation but also impacts biocompatibility, as excess aldehyde groups may irritate proteins/cells and impair cellular growth and proliferation (Moon and Pack, 1983). Consequently, successful fabrication of self-healing hydrogels via Schiff base covalent bonds requires optimized stoichiometric control of aldehyde group content.

### 4.2 Boronate ester bond

The boronate ester bond is a dynamic covalent bond formed through the reversible condensation reaction between boric acid (or its derivatives) and cis-1,2 or cis-1,3 diols. In recent years, boronate ester bonds have attracted significant attention in materials science, particularly in biomedical hydrogel design, due to their unique reversibility and environmental responsiveness. The empty p-orbital of the boron atom enables the formation of stable five- or six-membered cyclic structures with diol hydroxyl groups, allowing this reaction to proceed under mild conditions with high selectivity and tunability (Cambre and Sumerlin, 2011; Cromwell et al., 2015).

Boronate ester bonds exhibit high sensitivity to various external stimuli including pH variations, ROS and glucose concentration changes. Under pH stimulation, the stability of boronate ester bonds formed between boric acid and diols is closely related to solution acidity. Boric acid (or its derivatives) predominantly exists in two forms in aqueous solutions: neutral trigonal boric acid (sp^2^ hybridization) and negatively charged tetrahedral borate (sp^3^ hybridization), with their ratio determined by both the acid dissociation constant (pKa) and environmental pH. When pH < pKa, neutral boric acid dominates, facilitating nucleophilic reactions with diols to form boronate esters. Conversely, when pH > pKa, borate becomes the predominant species with reduced reactivity. Notably, the ionized borate ester (negatively charged) demonstrates superior stability and hydrolysis resistance compared to its neutral counterpart (Terriac et al., 2024). Although alkaline environments may reduce initial reaction rates, the resulting borate ester form significantly enhances overall stability. In contrast, neutral or weakly acidic conditions favor higher proportions of neutral boronate esters with accelerated hydrolysis rates, leading to material performance degradation. This inherent contradiction between reactivity and stability represents a critical challenge requiring careful balancing in current boronate ester-based material design.

Under oxidative stimulation, ROS such as H_2_O_2_ can attack boronic acid groups through deboronation reactions, resulting in hydrogel network disruption. This ROS sensitivity not only enables on-demand drug release in oxidative stress environments but also endows materials with ROS-scavenging capabilities to protect surrounding tissues from oxidative damage (Stubelius et al., 2019). Additionally, glucose can competitively bind with phenylboronic acid (PBA) through its 1,2-cis-diol structure, disrupting original hydrogel crosslinking networks. Under hyperglycemic conditions (e.g., diabetic wound microenvironments), glucose replaces diol ligands to form new boronate ester bonds with PBA, leading to reduced crosslinking density and volumetric swelling that enables controlled drug release. For instance, in diabetes treatment, glucose-mediated competitive binding in hyperglycemic environments facilitates on-demand insulin release, establishing closed-loop feedback regulation (Zhou et al., 2022).

The primary advantages of boronate ester hydrogels lie in their dynamic nature-derived multifunctionality, including self-healing properties, injectability, and precise responsiveness to multiple stimuli, making them broadly applicable in drug delivery, bioprinting, and cellular microenvironment simulation. However, limitations require attention: insufficient boronate ester stability under physiological pH (≈7.4) necessitates pKa reduction through molecular design (e.g., introducing ortho-amino groups or strong electron-withdrawing groups) to enhance binding affinity. Moreover, the dynamic equilibrium of boronate ester bonds is susceptible to competitive interference from polyhydroxy molecules in biological systems (e.g., natural sugars), causing non-specific degradation. The cytotoxic byproduct phenol generated from PBA deboronation further limits long-term implant applications.

### 4.3 Hemithioacetal bond

The hemithioacetal bond is a dynamic covalent bond formed through the thiol-aldehyde addition reaction, characterized by its structure containing both a hydroxyl group and a thioether group, with the general molecular formula R−CH(SR')−OH. This bond combines rapid crosslinking kinetics with mild reversible thermodynamic behavior, enabling spontaneous breakage-recombination cycles under physiological conditions (neutral pH, body temperature). Hemithioacetals exhibit faster reaction rates (k_1_ = 0.08–0.57 M^-1^s^-1^) and moderate equilibrium constants (K_eq = 3.8–72 M^-1^) due to the high polarizability and lower electronegativity of sulfur atoms, allowing controlled bond exchange even in mild environments (Lienhard and Jencks, 1966). These properties make hemithioacetal bonds a promising chemical module for constructing dynamic covalent networks, particularly suitable for biomaterial applications requiring rapid gelation while maintaining moderate reversibility.

A notable advantage of hemithioacetal bonds over traditional dynamic covalent bonds (e.g., boronate esters, Schiff base bonds) lies in their reduced pH dependence, enabling stable dynamic exchange under physiological neutral conditions and avoiding potential acidic damage to cells or tissues (Hua et al., 2019). Furthermore, the versatile reactivity of thiol groups offers enhanced functionalization and post-processing possibilities. For example, mild thiol-hemithioacetal exchange reactions with thiol-containing compounds (e.g., dithiothreitol, glutathione, or cysteine) facilitate gentle dressing removal (Yang et al., 2021b). Additionally, spontaneous conversion from dynamic to permanent crosslinking can be achieved via Michael addition with methacrylate groups, endowing hydrogels with initial operability and subsequent mechanical stability evolution (Yang et al., 2021a). In biomedical applications, this controllable crosslinking mechanism is particularly suited for wound dressings, tissue engineering scaffolds, and similar scenarios, minimizing tissue damage from secondary debridement while enhancing long-term functionality through post-curing.

However, hemithioacetal bonds also present limitations. First, despite their rapid kinetics, moderate equilibrium constants limit network mechanical strength, often necessitating secondary crosslinking strategies to meet high-load application requirements. Second, thiol groups are inherently sensitive to oxidation, as they can be oxidized by atmospheric or biological oxidants (e.g., ROS) into disulfide bonds (−S−S−), potentially disrupting the dynamic equilibrium and network integrity of hemithioacetals. Thus, design strategies must comprehensively consider reaction conditions, thiol protection approaches, and secondary network reinforcement to fully exploit the potential of hemithioacetal bonds in dynamic hydrogels.

### 4.4 Metal coordination crosslinking

Metal coordination crosslinking is a distinctive non-covalent interaction involving the formation of coordination bonds between ligands (via lone electron pairs) and vacant orbitals of metal ions. Its dynamic behavior lies intermediate between stable covalent bonds and weaker non-covalent interactions (e.g., hydrogen bonds, electrostatic forces). This mechanism is widely observed between biomolecules rich in coordination sites (e.g., proteins) and transition metal ions with unpaired electrons (e.g., Fe^3+^, Zn^2+^, or Cu^2+^) (Degtyar et al., 2014). Growing evidence highlights the critical role of metal coordination complexes in regulating the mechanical properties of biomaterials. For instance, marine mussels leverage metal coordination bonds to construct hierarchical material structures, achieving exceptional strength, elasticity, and toughness (Waite et al., 2005).

Metal coordination bonds uniquely endow materials with both high stiffness and high ductility—two properties traditionally considered mutually exclusive—thereby breaking the performance trade-off between rigidity and toughness in conventional materials. In engineered hydrogel systems, metal coordination bonds are often introduced as secondary networks within polymer backbones to enhance overall mechanical performance. Polyphenolic molecules such as 2,3,4-trihydroxybenzaldehyde (TBA) and dopamine can form stable yet dynamically reversible crosslinked networks with Fe^3+^ through monodentate, bidentate, or tridentate coordination modes (Teng et al., 2021; Peng et al., 2024).

The kinetics of metal coordination bonds (e.g., bond rupture-reformation rates) can be flexibly tuned by adjusting metal ion types, oxidation states, and ligand designs. This enables precise control over dynamic mechanical properties and response timescales without requiring polymer backbone resynthesis. Furthermore, metal ions exhibit migration capabilities within networks, allowing spontaneous relocation to new coordination sites or reversible “on-off” switching via chelating agents. This high programmability offers rich strategies for developing intelligent, renewable, and high-performance soft materials (Holten-Andersen et al., 2014).

Despite their unique advantages in dynamic reversibility, self-healing properties, and tunable mechanics, metal-coordinated hydrogels face several challenges. First, excessive metal ion usage may induce cytotoxicity or compromise biocompatibility, necessitating meticulous ratio optimization and release control. Second, coordination bond strength and dynamics are highly sensitive to environmental pH, temperature, and competing ions, risking network disintegration or performance drift in complex biological environments. Additionally, natural metal-coordinated networks in biological systems are intricately coupled with multilevel organizational architectures, posing significant challenges in mimicking their spatiotemporal dynamic regulation mechanisms in synthetic materials.

### 4.5 Weak non-covalent interactions

Weak non-covalent interactions—including hydrogen bonding, electrostatic interactions, hydrophobic effects, and π–π stacking—can form three-dimensional physically crosslinked networks with high dynamic reversibility and flexibility. These interactions, fundamental to supramolecular chemistry, are widely employed in the design and fabrication of dynamic hydrogels (Picchioni and Muljana, 2018). When hydrogel networks are disrupted by external forces or environmental factors, these weak non-covalent interactions rapidly re-establish, endowing hydrogels with self-healing capabilities.

Additionally, weak non-covalent bonds confer hydrogels with high sensitivity to external stimuli (e.g., pH, temperature, ionic concentration changes) and error-correction functionality, enabling materials to maintain structural and functional adaptability in dynamic environments. However, due to their inherently weaker nature compared to covalent bonds, hydrogels relying solely on weak non-covalent crosslinking typically exhibit significantly inferior mechanical properties (e.g., strength and elastic modulus) relative to chemically crosslinked hydrogels. To address this limitation, researchers have developed composite crosslinked networks by integrating weak non-covalent interactions with dynamic covalent bonds (e.g., Schiff base bonds), achieving an optimized balance between mechanical performance and functional responsiveness in materials (Han et al., 2022).

## 5 Biological functions of glycopeptide hydrogels

**FIGURE 1:**
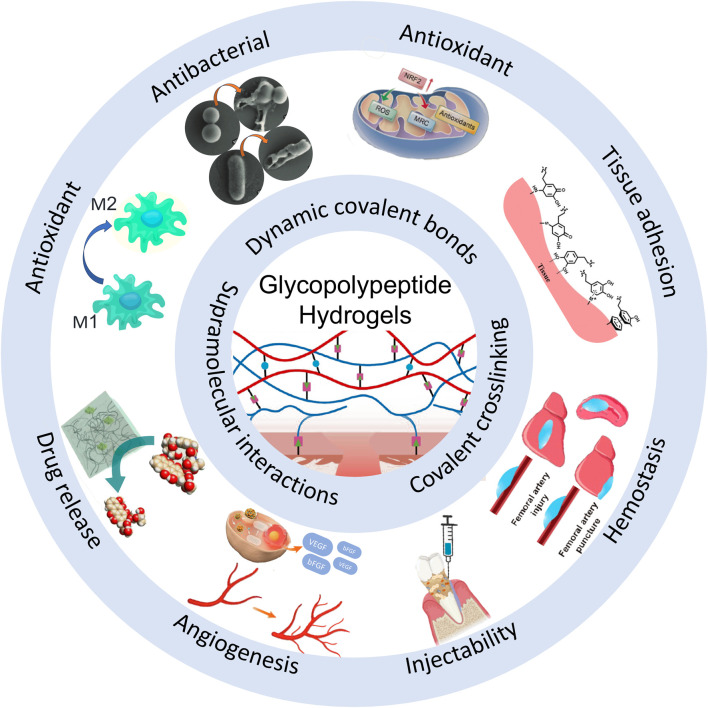
Schematic diagram of the biological functions of glycopeptide hydrogels. Antibacterial and angiogenesis (Wu et al., 2023), Copyright 2023. Reproduced with permission from John Wiley and Sons, Inc. Injectability (Zhao et al., 2024b), Copyright 2024. Reproduced with permission from the American Chemical Society. Hemostasis (Zhu et al., 2024), Copyright 2024. Reproduced with permission from the American Chemical Society. Drug release (Sakamoto et al., 2022), Copyright 2022. Reproduced with permission from the American Chemical Society. Antioxidant (Zhao et al., 2024a), Copyright 2023. Reproduced with permission from Elsevier B.V. Tissue adhesion (Teng et al., 2021), Copyright 2021. Reproduced with permission from John Wiley and Sons, Inc.

In the realm of biomedical materials, glycopeptide hydrogels have emerged as a groundbreaking solution with multifaceted biological functions. By leveraging the unique properties of glycopeptides, these hydrogels offer a versatile platform for addressing many challenges in wound healing, tissue regeneration, and drug delivery. The following sections delve into the intricate biological functions of glycopeptide hydrogels to explore their antibacterial properties, anti-inflammatory and immunoregulatory effects, antioxidant capabilities, drug delivery mechanisms, tissue adhesion, injectability, angiogenesis effects, and hemostatic potential (Figure 1). This comprehensive overview aims to highlight the significant advances and applications of glycopeptide hydrogels in modern biomedical science.

### 5.1 Antibacterial properties

Bacterial infections pose a major challenge in clinical wound healing, particularly in chronic wounds, surgical incisions, and implant-related infections. Persistent bacterial colonization and biofilm formation delay the healing process and may cause the wound to deteriorate. Hydrogels, with their excellent moisture-retention properties, provide an ideal healing environment for tissue. However, this same characteristic also promotes microbial proliferation, increasing the risk of infection (Wang et al., 2025a). Therefore, endowing glycopeptide hydrogels with effective antibacterial properties is a critical design requirement for their use in wound repair and tissue engineering. Currently, antibacterial strategies for glycopeptide hydrogels mainly include targeting multivalent glycan–lectin interactions, integrating antibacterial polymers, and loading the hydrogels with antimicrobial agents. While the first two approaches use non-antibiotic mechanisms that inherently minimize antibiotic resistance development, the third strategy provides supplementary antimicrobial action. Collectively, these methods effectively inhibit pathogens through distinct pathways, optimize the wound microenvironment, and promote healing and tissue regeneration.

#### 5.1.1 Targeting multivalent glycan–lectin interactions to inhibit antibacterial activity

**FIGURE 2:**
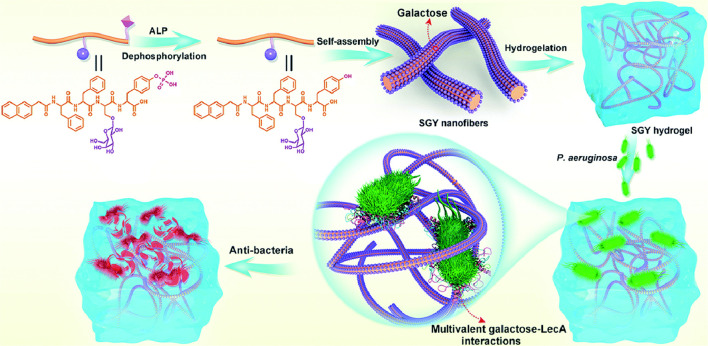
Schematic diagram of a multivalent sugar lectin-targeted antimicrobial hydrogel based on self-assembling peptides. (Liu et al., 2020), Copyright 2020. Reproduced with permission from the Royal Society of Chemistry.

Specific glycan and lectin interactions are widespread in biological systems and can be exploited to target bacterial surface lectins or other specific receptors to achieve precise antibacterial effects (Bernardi et al., 2013). However, the binding affinity between individual glycan molecules and lectins is relatively weak and often fails to exert a stable antibacterial effect (Cecioni et al., 2015; Miura et al., 2016). To overcome this limitation, a multivalent glycan-cluster approach was proposed, wherein multiple glycan molecules are densely arranged to significantly enhance their binding affinity to bacterial lectins (Imberty and Varrot, 2008). The self-assembling properties of glycopeptide hydrogels provide an ideal system for constructing supramolecular structures with multivalent glycan clusters to greatly strengthen glycan–lectin interactions and improve the antibacterial potency and specificity of the hydrogel. Using this strategy, hydrogels effectively capture bacteria, prevent adhesion and colonization, and inhibit bacterial growth without relying on antibiotics, and thus have high potential for targeted antibacterial applications (Li J. et al., 2019; Liu et al., 2020) (Figure 2).

#### 5.1.2 Integration of antibacterial polymers

Antibacterial polymers are high molecular-weight materials that autonomously inhibit bacterial growth through the following mechanisms:

(i) Electrostatic disruption of bacterial membranes: Certain cationic polymers (e.g., chitosan, EPL) possess positively charged groups that bind to anionic components (e.g., lipopolysaccharides in Gram-negative bacteria or teichoic acids in Gram-positive bacteria) on microbial membranes. This interaction disrupts membrane integrity, leading to leakage of cytoplasmic contents and bacterial lysis.
(ii) Interference with bacterial physiology: Beyond membrane disruption, some AMPs such as ILPWKWPWWPWRR can translocate into cells to inhibit vital processes such as DNA replication, protein synthesis, or cell wall assembly.

For example, Moon et al. (Moon et al., 2023) designed a glycopeptide hydrogel based on EPL and chitosan with sustained EPL degradation and release that significantly inhibited biofilm formation by multidrug-resistant *Pseudomonas aeruginosa*. This approach avoided the potential for antibiotic resistance and provided long-lasting antibacterial activity without relying on drugs, thus offering a safe and sustainable solution for eradicating bacterial infections.

#### 5.1.3 Antibacterial drug loading

In certain cases, directly loading antibacterial drugs into hydrogels provides strong antibacterial effects and controlled drug release. Glycopeptide hydrogels, with their porous structure and tunable degradation rates, serve as ideal carriers for antibiotics and antimicrobial molecules. For example, minocycline hydrochloride (MH), a broad-spectrum tetracycline antibiotic, has shown promise in treating periodontal infections, chronic wounds, and biofilm-associated infections. Studies showed that glycopeptide hydrogels loaded with MH continuously released the drug to effectively combat biofilm-encased resistant pathogens and reduced local inflammation and bacterial invasion risks (Zhao et al., 2024b). This type of hydrogel has demonstrated excellent antibacterial effects in treating periodontal disease, chronic wounds, and surgical implant infections.

Beyond traditional antibiotics, emerging strategies such as photothermal sterilization and ionic interference have been successfully integrated into glycopeptide hydrogels. For instance, Shen et al. (Shen et al., 2024) designed a glycopeptide hydrogel (deferoxamine/CuS-ECMgel) by crosslinking HA with RGD peptides and matrix metalloproteinase degradable peptides. The hydrogel encapsulated CuS nanoparticles and deferoxamine, achieving dual functions of photothermal antibacterial activity and angiogenesis promotion. Under near-infrared irradiation, CuS nanoparticles generated localized heat to eradicate *Escherichia coli* and Methicillin-resistant *Staphylococcus aureus* with 99% efficiency. Ma et al. (Ma et al., 2024) developed an injectable glycopeptide hydrogel (Ag@ZnO/G-H), combining gelatin methacrylate (G) and methacrylate modified HA (H) to form a biomimetic network. This hydrogel embedded with Ag@ZnO heterojunction nanoparticles achieved dual antibacterial modes: (i) sustained release of Ag^+^/Zn^2+^ ions disrupting bacterial membranes, and (ii) visible light-triggered ROS generation via enhanced electron-hole separation. The “ROS storm” eradicated 99% of drug-resistant bacteria (e.g., Methicillin-resistant *S. aureus*).

These studies highlight the potential of combining glycopeptide hydrogels with photothermal or ionic components to achieve multifunctional antibacterial effects, offering alternatives to conventional antibiotics and minimizing resistance development.

### 5.2 Anti-inflammation and immunoregulation

The inflammatory response is the core protective mechanism by which the body responds to external stimuli, removes pathogenic microorganisms, and repairs damaged tissue. This process is driven by the activation of immune cells and their secretion of pro-inflammatory factors, such as TNF-α and interleukin-6 (IL-6), and anti-inflammatory factors such as interleukin-10 (IL-10). However, excessive or prolonged inflammation caused by bacterial infections, physical and chemical damage, and metabolic abnormalities can produce tissue destruction, an immune imbalance, and even fibrosis, all of which significantly hinder tissue repair (Zhao et al., 2020; Pahwa et al., 2025). Therefore, intervening appropriately to accelerate tissue repair in an inflammatory microenvironment has become a key aspect of regenerative medicine (Li J. et al., 2021; Villarreal-Leal et al., 2021). A critical factor in immunoregulation is the precise control of the macrophage polarization balance and immune cell crosstalk to enhance the expression of anti-inflammatory factors and restore immune homeostasis (Xiong et al., 2022; Brown et al., 2012).

Glycopeptide hydrogels regulate the inflammatory microenvironment and promote tissue repair through their anti-inflammatory and immunoregulatory properties. Their anti-inflammatory action effectively limits the intensity and scope of the inflammatory response by directly inhibiting the release of inflammatory factors and related signaling pathways. Immunoregulation primarily functions by inducing macrophage polarization or enhancing T-cell and macrophage crosstalk to reshape the immune microenvironment into a reparative state. Specifically, by leveraging the innate immunoregulatory properties of natural polysaccharides or loading bioactive molecules or anti-inflammatory drugs into glycopeptide hydrogels, excellent anti-inflammatory and immunoregulatory effects can be achieved (Table 4).

**TABLE 4:** Anti-inflammatory and immunomodulatory substances in glycopeptide hydrogels: Sources and mechanisms.

Category	Substance	Source	Mechanism
Polysaccharides	Hyaluronic acid	Found in synovial fluid, skin, and other human tissues	Binding to cell surface receptors such as CD44, and inhibiting the release of inflammatory factors (Marinho et al., 2021)
	Chitosan	Derived from the shells of crustaceans such as shrimp and crabs	Modulating the maturation, activation, cytokine production, and polarization of dendritic cells and macrophages (Ghattas et al., 2025)
	Alginate	Extracted from brown algae	Reducing the serum concentrations of pro-inflammatory cytokines TNF-α and IL-6 (Lukova et al., 2024)
	Fucoidan	Extracted from brown algae and other seaweeds	Acting on different stages of the inflammatory process: blocking of lymphocyte adhesion and invasion, inhibition of multiple enzymes, and induction of apoptosis (Apostolova et al., 2020)
	Glucomannan	Extracted from certain plant species and fungi	Activating mannose receptors, induces M2 macrophage polarization, and inhibiting the release of pro-inflammatory factors (Pan et al., 2024)
	Dextran	Produced through microbial fermentation	Stimulating the proliferation of murine macrophages and decreasing the release of nitric oxide by the cells (Soeiro et al., 2016)
	*Bletilla striata* polysaccharide	Derived from *Bletilla striata* (type of orchid)	Exhibiting anti-inflammatory and immunomodulatory effects by activating NF-κB and MAPK signaling pathways, enhancing macrophage phagocytosis and cytokine secretion (Niu et al., 2022)
Bioactive substances	Paeoniflorin	Root of *Paeonia lactiflora* (peony)	Regulating immune cells, enhancing anti-inflammatory mediators (Akt, PKA, IL-4, IL-10, TGF-β), inhibiting pro-inflammatory factors (COX-2, IL-1β, IL-6, IL-17, IFN-γ), and modulating GPCRs, NF-κB, MAPK, and PI3K/Akt pathways (Zhou et al., 2020)
	Astragaloside IV	*Astragalus membranaceus*	Modulating inflammatory factors (IL-1β, TNF-α, ICAM, chemokines), inflammatory mediators (NO), the NF-κB pathway, and apoptosis-related genes to counteract inflammatory damage (Liang et al., 2023)
	Salvianolic acid B	*Salvia miltiorrhiza* (danshen)	Inhibiting NF-κB activation, reducing TNF-α expression, suppressing inflammatory cell infiltration, and downregulating adhesion molecules (VCAM-1, ICAM-1) (Wang and Zeng, 2019)
	Epigallocatechin-3-gallate	Green tea	Regulating T cell balance, inhibiting pro-inflammatory cytokines (TNF-α, IL-6, IL-1β), suppressing NF-κB and Jak/STAT pathways, and reducing immune cell migration (Zhao et al., 2021)
Cell-derived bioactive factors	Extracellular vesicles	Various sources	Downregulating cytokines, chemokine signaling pathways, and Toll-like receptor signaling; regulating immune responses by promoting macrophage polarization from the M1 to M2 phenotype (Huang et al., 2025)
Small molecule drugs	Aspirin	Synthetic	Inhibiting COX activity, reducing the production of inflammatory mediators such as prostaglandins (Hussain et al., 2012)
	Diclofenac sodium	Synthetic	Competing with arachidonic acid for binding to cyclo-oxygenase, resulting in decreased formation of prostaglandins (Small, 1989)

Furthermore, regulating the pH of the microenvironment has been shown to promote macrophage polarization toward the M2 phenotype (Xia et al., 2023). It is noteworthy that, in addition to macrophages, reparative TH2-type immune responses also promote tissue repair. In a study by Wang et al. (Wang J. et al., 2024), a glycopeptide hydrogel based on GM and the branching T-cell epitope peptide R4K4 enhanced immune crosstalk between macrophages and T cells. The hydrogel significantly restored tissue structure and promoted hair follicle regeneration by stimulating Th2 immune responses, increasing M2 macrophage recruitment, and promoting local angiogenesis.

### 5.3 Anti-oxidative stress

Oxidative stress refers to the pathological phenomenon in which ROS or reactive nitrogen species (RNS) are excessively produced, surpassing the body’s antioxidant defense capabilities, and culminating in cellular and tissue damage. ROS cause lipid peroxidation, DNA damage, and protein denaturation, which directly impair cell functions and activate pro-inflammatory signaling pathways (e.g., NF-κB and Nrf2) (Muro et al., 2024), exacerbating inflammation and creating a vicious cycle between inflammation and oxidative stress. Oxidative stress is considered a key pathogenic factor in many chronic diseases, including neurodegenerative diseases, metabolic disorders, and inflammation-related diseases (e.g., periodontitis and inflammatory bowel disease) (Jomova et al., 2023). Therefore, treatments must be developed that efficiently eliminate ROS and restore the cellular redox balance to reduce oxidative damage and prevent disease progression.

The use of natural polysaccharides, such as *Bletilla*, fucoidan, alginate, and kelp polysaccharides, and peptides such as KYKYEYEY and DOPA_4_-G_4_-GRGDS with antioxidant activity provides a simple means of designing effective glycopeptide hydrogels. Loading or conjugating hydrogels with polyphenolic substances such as tannic acid, GA, EGCG, and TBA is also a common approach. Polyphenolic substances scavenge free radicals through hydrogen atom transfer and electron donation and provide additional anti-inflammatory and antibacterial functions. It is noteworthy that due to their unique structural properties, polyphenols form multiple interactions with peptides and polysaccharides and chelate with metal ions to form a secondary network; this, in turn, can enhance the mechanical properties and tissue adhesion of the glycopeptide hydrogel (Zhang et al., 2024; Peng et al., 2024). Manganese dioxide decomposes hydrogen peroxide (H_2_O_2_) into oxygen in an acidic environment and releases Mn^2+^ to participate in metabolism. It has also been used to alleviate oxidative stress and improve wound hypoxia (Wu et al., 2023). In addition to the aforementioned substances, vitamin C, glutathione, arginine, cysteine, selenium, and natural enzymes have demonstrated satisfactory efficacy in treating various chronic diseases induced by ROS. Building on this foundation, Wang et al. (Wang et al., 2025b) designed a glycopeptide hydrogel embedded with selenocysteine-functionalized microspheres. The selenocysteine continuously scavenges excessive H_2_O_2_ expressed in the cellular microenvironment, enhances selenoprotein expression levels in the short term, and ultimately directly modulates the expression of thioredoxin reductase and glutathione peroxidase in cells, thereby maintaining their direct antioxidant functions and regulating redox balance.

In addition, boronic ester bonds and thioketal bonds exhibit degradation characteristics in high ROS environments and help scavenge ROS during the degradation process, thus providing an auxiliary antioxidant effect. Deng et al. (Deng et al., 2024) designed smart glycopeptide hydrogels based on these two chemical bonds to precisely release the encapsulated natural antioxidant astragaloside IV(AST) in high ROS microenvironments, synergistically improving the oxidative microenvironment of chronic wounds.

### 5.4 Drug delivery and responsive release

Glycopeptide hydrogels are highly valuable in drug delivery systems due to their dynamic three-dimensional network structure, high hydrophilicity, degradability, and biocompatibility. Glycopeptide groups of a hydrogel mimic the specific binding of natural sugar molecules to cell surface receptors, enabling highly targeted drug delivery. The dynamic crosslinking and self-healing properties of glycopeptide hydrogels maintain structural integrity during drug release, supporting long-term sustained release. Glycopeptide hydrogels have a structure influenced by both polysaccharides and peptides, with multiple supramolecular interactions and dynamic covalent bonds that can stably load different types of drugs (e.g., aspirin, docetaxel, minocycline, vancomycin, and deferoxamine), biomacromolecules (e.g., cordycepin), cell-derived components (e.g., extracellular vesicles, EVs), enzymes (e.g., β-galactosidase) or polymer microspheres (e.g., polylactic acid microspheres (Wang et al., 2025b)). Moreover, the pore size, mechanical strength, and degradation characteristics of glycopeptide hydrogels can be regulated, providing design flexibility and adaptability to meet different drug delivery needs.

**FIGURE 3:**
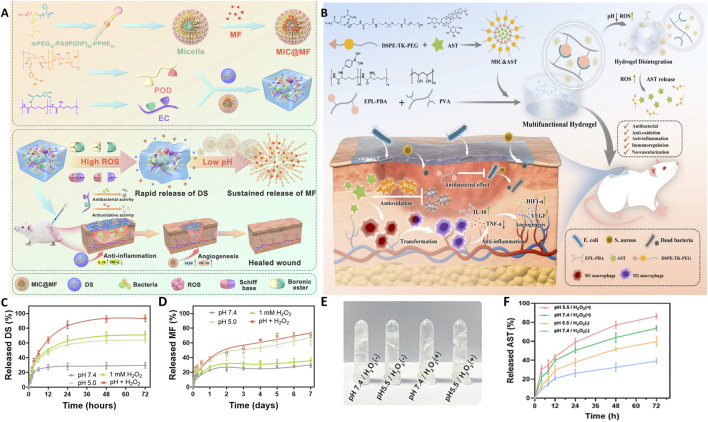
Schematic illustrations and release profiles of smart glycopeptide hydrogels used for targeted drug delivery and wound healing. **(A)** Schematic illustration of the pH/ROS dual-responsive injectable glycopeptide hydrogel DS&MIC@MF. **(B)** Schematic illustration of the smart responsive hydrogel EPBA-PVA@MIC&AST. **(C,D)** Release kinetics of DS **(C)** and MF **(D)** from the DS&MIC@MF hydrogel under different conditions. (Wu et al., 2022), Copyright 2021. Reproduced with permission from Elsevier B.V. **(E,F)** pH/ROS dual-responsive behavior **(E)** and AST release kinetics **(F)** of the EPBA-PVA@MIC&AST hydrogel. (Deng et al., 2024), Copyright 2023. Reproduced with permission from Elsevier B.V. AST: astragaloside IV; DS: diclofenac sodium; EPBA: Phenylboronic acid grafted ε-Poly-L-lysine; MF: mangiferin; MIC: micelles; PVA: polyvinyl alcohol.

By leveraging these features, smart hydrogels with multiple response characteristics and sequential drug release have been designed. Wu et al. (Wu et al., 2022) developed a pH/ROS dual-responsive injectable glycopeptide hydrogel using phenylboronic acid-grafted oxidized dextran and caffeic acidgrafted EPL. pH-responsive micelles (MIC) encapsulating mangiferin (MF) (MIC@MF) and diclofenac sodium (DS) were embedded in the hydrogel (Figure 3A). Under acidic and oxidative conditions, Schiff base and boronic ester bonds hydrolyzed, rupturing the hydrogel network and exposing DS and MIC@MF to the external solution. DS was rapidly released in the initial phase (release rate reached 84.5% within 24 h), while MIC-encapsulated MF was slowly and continuously released over 7 days (Figures 3C,D). This spatiotemporal delivery behavior aligned closely with the programmed process of infected wound healing (Rodrigues et al., 2019). Similarly, Deng et al. (Deng et al., 2024) prepared an EPBA-PVA@MIC&AST smart responsive hydrogel, where EPBA (PBA grafted EPL) was synthesized by conjugating PBA to EPL, and then polyvinyl alcohol was grafted onto the EPBA to form the EPBA-PVA hydrogel. This hydrogel has pH and ROS dual-responsive properties and encapsulates AST-loaded micelles (MIC&AST). AST was continuously released for 72 h, promoting sustained angiogenesis (Figures 3B,E,F) (Deng et al., 2024).

### 5.5 Tissue adhesion

The strong interfacial adhesion between hydrogels and tissue is one of the key factors ensuring the stability and reliability of the hydrogel functionality in various biomedical applications (Zhang W. et al., 2020; Zhang C. et al., 2020; Ma et al., 2021). However, tissue adhesion is challenging to achieve, mainly due to the complexity of both the internal and external environments of hydrogels. The high water content of hydrogels produces weak boundary layer effects and reduces surface energy (Cui and Liu, 2021; Hofman et al., 2018). Furthermore, the dynamic nature and the chemical and mechanical complexity of biological tissues further limit strong adhesion (Bhagat and Becker, 2017; Bouten et al., 2014). These challenges require the use of multiple material design strategies, such as dynamic covalent bonds, mussel-inspired chemical mechanisms, and supramolecular interactions, to overcome interfacial weaknesses and achieve stable and durable wet tissue adhesion.

In glycopeptide hydrogels, tissue adhesion is typically achieved by modifying the surface chemistry and optimizing the network structure of the hydrogel. A classic means of enhancing adhesion under wet conditions is covalent crosslinking between aldehyde and amine groups via the Schiff base reaction. When imine bonds in hydrogels break, aldehyde groups are exposed, which then react with amine groups on the tissue surface to form new imine bonds, thereby enhancing tissue adhesion (Huang et al., 2025; Matsumura et al., 2014). Semiquinone bonds also demonstrate excellent tissue adhesion properties (Yang et al., 2021b).

The introduction of natural polysaccharides, such as chitosan, and functional groups, such as catechols, tannic acid, GA, and dopamine, further strengthens adhesion. Chitosan, which carries a positive charge under physiological conditions, forms ionic and covalent bonds with negatively charged components on the tissue surface, thus enhancing adhesion and stability (Pan et al., 2025). Catechol groups, inspired by mussels, undergo partial deprotonation and transformation into reactive o-dihydroxyphenyl groups under physiological conditions, which then interact via covalent and non-covalent bonds with amine, imidazole, and thiol groups on the biological matrix surface to achieve tissue adhesion (Waite et al., 2005; Shi et al., 2023). Adhesion molecules such as RGD peptides (Zhao et al., 2024a; Lavrador et al., 2020) and galactose (Proksch et al., 2024), when incorporated into glycopeptide hydrogels, mimic the natural adhesion mechanisms of the extracellular matrix to promote efficient tissue integration.

### 5.6 Injectability

Injectable hydrogels are a class of materials that can be delivered to target sites within the body by injection, such as through needles and catheters, to form gels *in situ*. The core characteristics of these materials are their shear-thinning behavior and excellent mechanical stability. Their shear-thinning behavior allows the hydrogels to liquefy under shear stress in needles and catheters for delivery; once the stress is relieved, they rapidly recover their original structure and mechanical properties due to their dynamic crosslinked network. This phenomenon is produced by the disruption and reconstruction of dynamic covalent bonds and non-covalent interactions such as hydrogen bonds, hydrophobic interactions, and π–π stacking within the hydrogel (Bertsch et al., 2023).

Injectable hydrogels meet the urgent needs of modern medicine to provide minimally invasive treatments and precision medicine, particularly in parts of the body with complex anatomical structures or those that are difficult to access surgically, such as the spinal cord, heart, joint cavities, and vitreous cavity. Compared with traditional implanted hydrogels, injectable hydrogels significantly reduce wound size and postoperative complications, shorten recovery time, and provide precise treatment through local drug delivery and mechanical support. The challenge of these hydrogels is how to balance their flowability and structural stability to ensure smooth and efficient delivery during injection while still achieving rapid recovery and maintenance of a stable structure and its function after injection, thus ensuring long-lasting and effective performance at the target site (Dodda et al., 2024; Mo et al., 2024).

Glycopeptide hydrogels are a unique material that fully leverages the dynamic crosslinking characteristics of glycosyl and peptide chains in their design, which easily achieves the required injectability (Omidian et al., 2024). Injectable glycopeptide hydrogels are used for minimally invasive delivery in complex anatomical locations and have shown application potential in tissue regeneration and precision therapy. For example, in a porcine myocardial infarction model, a decellularised ECM (dECM)/glycopeptide(GP) composite hydrogel was injected into the myocardium, where it rapidly gelled, with a stiffness of approximately 1,000 Pa. This provided ECM stability and facilitated cell infiltration and angiogenesis (Kong et al., 2023). In central nervous system injuries, a refrigerated CRP hydrogel, composed of chitosan, RADA16 and PPFLMLLKGSTR peptide, injected via minimally invasive means effectively filled irregular spinal cord injury tissue and rapidly solidified at body temperature as a scaffold. The formed glycopeptide hydrogel bridged the spinal cord injury and promoted nerve regeneration (Sun et al., 2024). In cartilage tissue repair, the local application of an oxidized dextran/carboxymethyl chitosan/EPL/synovial mesenchymal stem cell-derived EVs (OD/CS-PL@EVs) glycopeptide hydrogel via injection into torn menisci filled defects *in situ* and promoted the reintegration of the torn menisci (Huang et al., 2025).

### 5.7 Angiogenesis

Glycopeptide hydrogels have demonstrated significant application potential in angiogenesis, which is of profound significance for treating chronic wounds and ischemic diseases, as well as for tissue regeneration. The formation of new blood vessels is a key step in wound healing and tissue regeneration, as the new vessels provide oxygen and nutrients, act as a scaffold for ECM remodeling and cell migration, accelerate waste clearance, and resolve inflammation (Chen et al., 2023; Li Y. et al., 2021). However, in chronic wounds and ischemic microenvironments, vascular occlusions and hypoxia are major obstacles. Glycopeptide hydrogels, with their unique biomimetic functions and bioactivity, provide a new approach to addressing this issue. Common glycopeptide hydrogel strategies include promoting M2 macrophage polarization by introducing natural polysaccharides or using glycosyl modifications such as mannose, enhancing local immune regulation, and secreting angiogenic factors such as VEGF. Self-assembled nanofibers and porous structures also enhance endothelial cell adhesion, migration, and lumen formation (Wang J. et al., 2024; Wang et al., 2022; Feng et al., 2020; Proksch et al., 2024). In addition, the drug-loading and controlled release capabilities of hydrogels, such as AST or deferoxamine, further promote endothelial cell proliferation and new capillary formation (Qi et al., 2018; Deng et al., 2024). Recent studies showed that by regulating signaling pathways, such as PI3K/Akt and the downstream VEGF pathways MAPK, RAP1, and RAS, glycopeptide hydrogels maintained their angiogenic capacity in adverse environments, such as hypoxic and radiation-induced damaged tissue (Guo et al., 2024; Wu et al., 2023; Kong et al., 2023). These properties enable glycopeptide hydrogels to be multifunctional and controllable materials that can provide innovative solutions for chronic wound healing and tissue repair.

### 5.8 Hemostasis

Glycopeptide hydrogels, as novel hemostatic materials, have multiple advantages and provide innovative solutions for treating complex bleeding scenarios and acute trauma. Their excellent performance is closely tied to achieving an optimized design. For example, by precisely controlling the microporous structure, a hydrogel can rapidly adsorb blood and concentrate coagulation cells, significantly increasing the hemostatic efficiency (Teng et al., 2021). In addition, cationic groups such as–NH_3_^+^ in the material effectively adsorb negatively charged platelets and red blood cells, which significantly accelerates the clotting process (Peng et al., 2024). The introduction of catechol groups enhances the adhesion of the hydrogel in a moist tissue environment, allowing it to firmly adhere to a wound surface. Glycopeptide hydrogels have low hemolysis rates and good tissue compatibility, supporting their potential for wound healing applications. In addition to rapid hemostasis, glycopeptide hydrogels also regulate the wound microenvironment to support healing.

**FIGURE 4:**
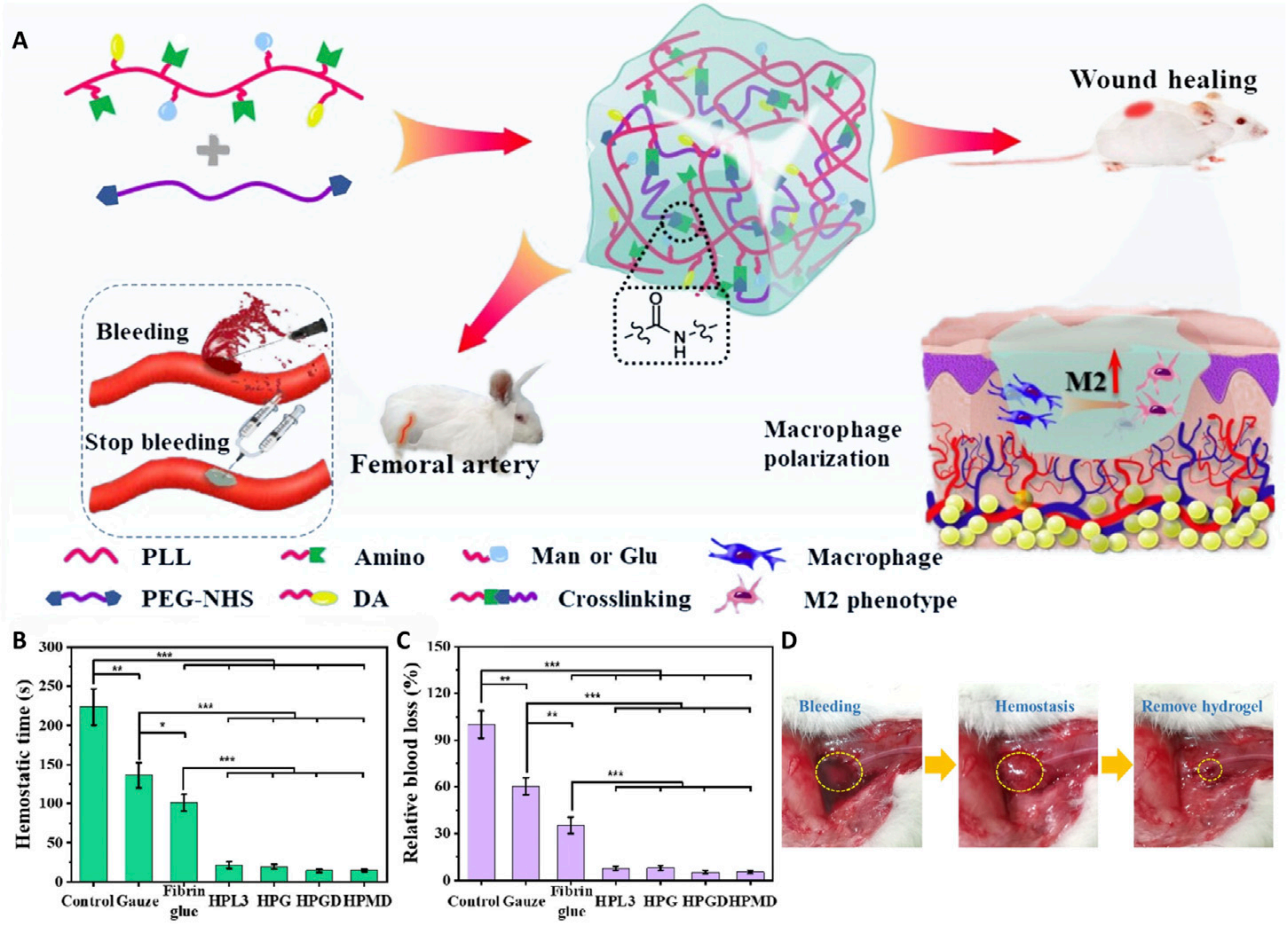
Application of a glycopeptide hydrogel in hemostasis. **(A)** Schematic illustration of the preparation and application of a biomimetic PEGylated glycopeptide hydrogel that modulated the inflammatory wound microenvironment to promote arterial hemostasis and wound healing. **(B,C)** *In vivo* hemostatic performance of the hydrogel in a rat femoral artery model. Relative blood loss **(B)** and hemostasis time **(C)** for different hydrogel formulations. **(D)** Representative photographs demonstrating the hemostatic effect of the hydrogel: (left) initial bleeding, (middle) hemostasis achieved with the hydrogel, and (right) hydrogel removal after hemostasis. (Teng et al., 2024), Copyright 2024. Reproduced with permission from the American Chemical Society.

Leveraging these properties, Teng et al. (Teng et al., 2024) designed a polyethylene glycol-modified glycopeptide hydrogel with a burst pressure resistance of up to 150 mmHg that was able to withstand high-pressure blood flow environments and achieve rapid hemostasis in less than 20 s (Figures 4B, C). The hydrogel was also modified with mannose, which effectively induced macrophage polarization to the anti-inflammatory M2 type and inhibited the expression of pro-inflammatory factors, such as TNF-α, which significantly alleviated wound inflammation (Figure 4A). This design achieved rapid hemostasis and promoted dermal regeneration, a high-density hair follicle distribution, and 90% collagen deposition within 14 days, demonstrating remarkable healing potential. In addition, the hydrogel was easily removed within 5 min after hemostasis and prevented secondary damage and bleeding, thus providing a safe and convenient treatment for emergency situations such as massive arterial bleeding (Figure 4D).

## 6 Glycopeptide hydrogels in organ engineering

### 6.1 Glycopeptide hydrogel skin applications

The skin is the largest organ of the human body and the first line of defense but is easily affected by mechanical, chemical, and radiation injuries; surgical operations; and chronic diseases such as diabetes. Wound healing involves four stages, hemostasis, inflammation, proliferation, and remodeling, all of which rely on the coordinated action of various cell types and the ECM (Blacklow et al., 2019; Guo and DiPietro, 2010). However, factors such as aging, immune deficiencies, and peripheral arterial disease significantly impair skin regeneration, allowing chronic hard-to-heal wounds to develop. These wounds are often characterized by fibroblast and endothelial cell dysfunction, excessive inflammation, elevated ROS levels, and bacterial infections, and ultimately fall into a vicious cycle marked by microenvironmental imbalances (Chopra et al., 2022). Breaking this cycle requires increased oxygenation, elimination of ROS, inhibited inflammation, and improved angiogenesis (Wu et al., 2023).

In recent years, glycopeptide hydrogels, as biomimetic materials, have shown strong potential for accelerating skin wound healing due to their excellent biocompatibility, biodegradability, and multifunctionality. The core advantage of glycopeptide hydrogels lies in their ability to mimic the glycoprotein components and nanofiber structures of the ECM. This improves the wound microenvironment and promotes tissue repair and regeneration through multiple mechanisms, including ROS elimination, macrophage polarization regulation, inflammation suppression, and angiogenesis. Because of these properties, glycopeptide hydrogels are broadly used in chronic hard-to-heal wounds, diabetic wounds, skin radiation injuries, and other conditions. Nevertheless, challenges persist in matching degradation rates to the prolonged healing timeline of chronic wounds and achieving sufficient mechanical strength for high-tension areas (e.g., joints), where rapid structural failure may occur under cyclic stress.

**FIGURE 5:**
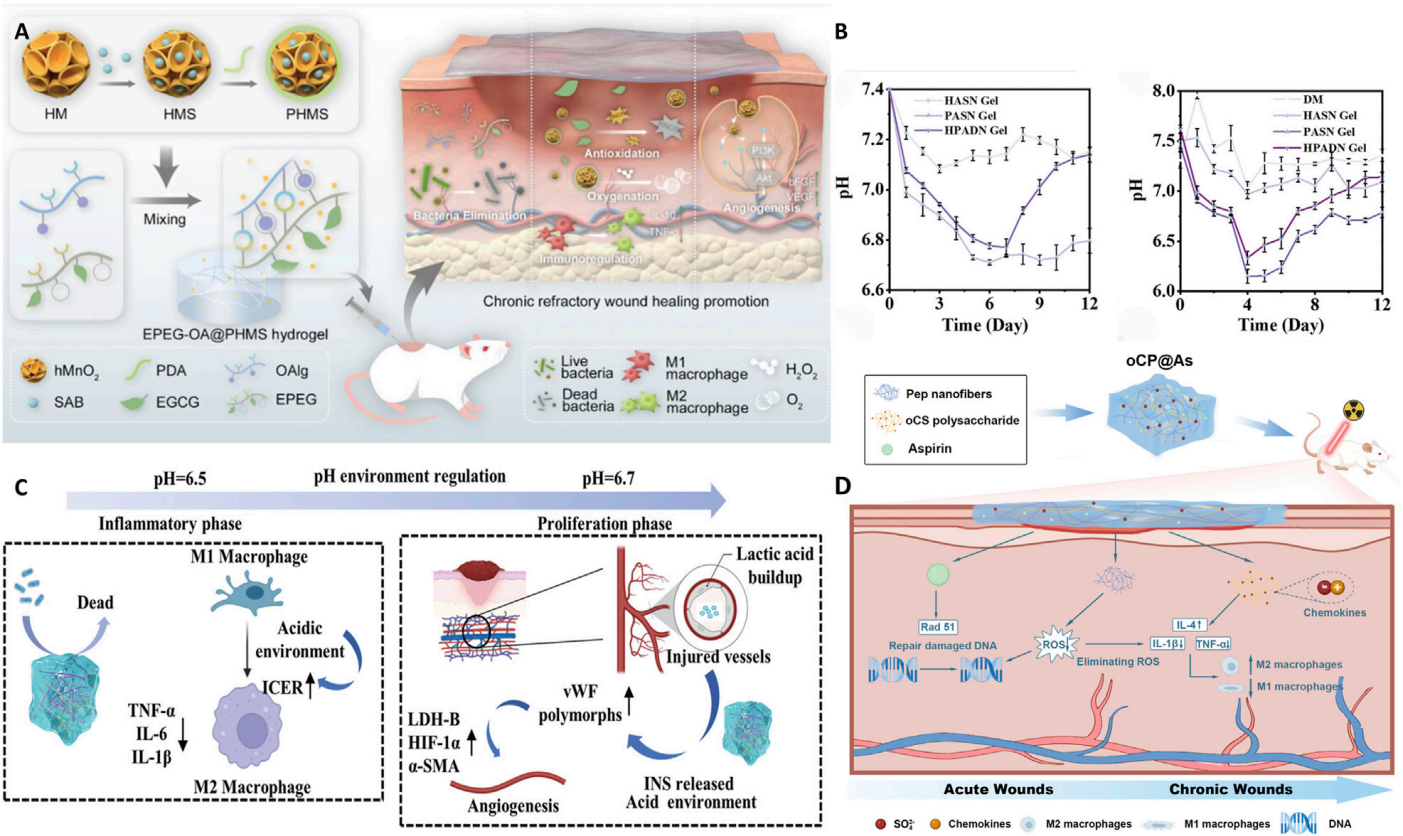
Application of a glycopeptide hydrogel in the skin. **(A)** Schematic diagram of the preparation method and wound healing mechanism of the EPEG-OA@PHMS hydrogel. (Wu et al., 2023), Copyright 2023. Reproduced with permission from John Wiley and Sons, Inc. **(B,C)** Dynamic pH-regulated wound healing promoted by the HPADN hydrogel at different stages. HPADN hydrogel included HA modified with diacylhydrazine adipate (HA-ADH) or aldehyde (OHA), and dopa-modified poly(6-aminohexanoic acid) (PADA). (Liu et al., 2024), Copyright 2024. Reproduced with permission from Elsevier Science, Ltd. **(D)** Dual-phase regulatory strategy of the oCP@As hydrogel. (Guo et al., 2024), Copyright 2024. Reproduced with permission from the American Chemical Society. EPEG-OA: phenylboronic acid-grafted EPL, epigallocatechin-3-gallate and oxidized alginate; PHMS: resveratrol B-loaded, honeycomb-like MnO2 nanoparticles.

To meet the dynamic and complex requirements for healing chronic wounds, Wu et al. (Wu et al., 2023) prepared a multifunctional glycopeptide hydrogel consisting of phenylboronic acid-grafted EPL (EPBA), EGCG, and oxidized alginate. The hydrogel incorporated custom-made polydopamine-coated, herb-derived, resveratrol B-loaded, honeycomb-like MnO_2_ nanoparticles (PHMS). The hydrogel dissociated in response to pH and ROS, which released PHMS nanoparticles that effectively cleared ROS and RNS. and continuously generated oxygen, thus maintaining the intracellular redox balance. In addition, the hydrogel encapsulated with resveratrol B regulated macrophage polarization, alleviated inflammation, and promoted angiogenesis by activating the PI3K/Akt pathway, thereby comprehensively accelerating skin repair through multiple stages of the wound healing process (Figure 5A). The hydrogel demonstrated significant therapeutic effects in a diabetic skin wound model infected with *Staphylococcus aureus* in Sprague–Dawley rats, including bacterial eradication, oxidative stress improvements, inflammation reductions, new blood vessel formation, and collagen deposition. Similarly, Zhang et al. (2024) and Deng et al. (2024) constructed hydrogels with pH and ROS dual-responsive characteristics. By embedding herbal extracts into ECM-mimicking hydrogel networks, they enhanced the antimicrobial and antioxidant properties of the hydrogel while optimizing the treatment effects during the inflammation, proliferation, and remodeling stages, which accelerated the healing of chronic hard-to-heal wounds.

In diabetic wound management, glycopeptide hydrogels based on biomimetic ECM structures that can intelligently regulate the microenvironment have become an important research direction for diabetic wound repair. Liu et al. (Liu et al., 2024) designed a hydrogel comprising oxidized glucomannan and functional peptides, which formed a network structure through Schiff base and hydrogen bond interactions. The hydrogel exhibited self-healing, antibacterial, and antioxidant properties, significantly promoting angiogenesis and tissue regeneration in diabetic wounds by promoting the M2 macrophage phenotype. Xia et al. (Xia et al., 2023) designed a novel hydrogel (HPADN) using HA modified with diacylhydrazide hexanedioate (HA-ADH) or aldehyde (OHA) and dopamine-modified poly-6-aminohexanoic acid (PADA). The hydrogel dynamically regulated pH through the release of H^+^ release by PADA and the capture of H^+^ by HA-ADH (Figure 5B). The regular changes in pH (alkaline–acidic–neutral) met the pH requirements of the wound microenvironment during different healing stages and promoted tissue regeneration by regulating immune cell (macrophages and endothelial cells) behavior, providing an innovative strategy for diabetic wound treatment (Figure 5C).

In radiodermatitis (RSI), in addition to acute damage caused by excessive ROS and chronic inflammation induced by macrophage homeostasis imbalance, radiation also induces the DNA damage response, with persistent or irreparable DNA damage, irreversible cell cycle arrest, and the release of senescence-associated secretory phenotype factors. These factors ultimately lead to cell senescence and stagnated proliferation, which affect the function of surrounding normal cells and hinder wound healing (Bloom et al., 2023). Feng et al. (Feng et al., 2023) used GM, polymerized peptides (K2(SL)6K2), and tannic acid to generate antioxidant activity, which enhanced tissue adhesion and regulated inflammatory microenvironment functions, significantly reducing radiation-induced acute damage and chronic inflammation. Guo et al. (Guo et al., 2024) designed a biomimetic self-assembled glycopeptide hydrogel (oxidized chondroitin sulfate-peptide@aspirin, oCP@As) using dual-phase regulation (early-stage ROS scavenging, DNA repair, and late-stage inflammation mediation) (Figure 5D) for on-demand treatment of RSI. The hydrogel featured an ECM-mimetic nanofiber structure and incorporated oxidized chondroitin sulfate to regulate the inflammatory response. It remodeled the inflammatory microenvironment and demonstrated excellent anti-inflammatory and repair effects during both the acute and chronic phases of wound repair. Wang et al. (Wang H. et al., 2024) prepared a glycopeptide hydrogel with self-assembled peptides (Nap-FFRR) and HA, in which encapsulated cordycepin was released in a controlled manner through dynamic covalent binding. The hydrogel effectively inhibited radiation-induced cellular senescence, while the arginine in the peptide chain provided antioxidant properties and acted as a nitric oxide (NO) precursor to promote angiogenesis. Animal experiments demonstrated that the hydrogel significantly improved the repair of radiotherapy-damaged tissue, reduced cellular damage, and accelerated wound healing, thus offering an innovative solution for wound management after neoadjuvant radiotherapy.

As an anti-aging treatment for skin, glycopeptide hydrogels exhibit outstanding hydration and microenvironment regulation capabilities. Zhang et al. (Zhang et al., 2025) developed a glycopeptide hydrogel (γ-PGA/HA) comprising γ-PGA and HA in the form of microneedle patches. The hydrogel effectively penetrated the skin barrier to provide significant moisturizing and fibroblast-promoting effects, ROS consumption, and TNF-α inhibition, with high biocompatibility. This hydrogel exhibited the potential for regulating the skin microenvironment and supporting applications in skin aging treatment.

### 6.2 Glycopeptide hydrogel applications in bone and cartilage repair

Bone and cartilage tissue damage presents significant clinical challenges, particularly because of their limited self-repair capacity, and often leads to irreversible tissue degeneration. Traditional treatment methods, such as artificial implants or growth factor delivery systems, can promote repair to some extent but face issues such as insufficient biocompatibility, long-term integration challenges, and immune rejection. Therefore, in recent years, bioactive scaffolds based on glycopeptide hydrogels have become a research focus of bone and cartilage regenerative medicine. The ECM-mimicking characteristics, immune regulation capabilities, and degradability of glycopeptide hydrogels may offer superior therapeutic results in bone and cartilage repair. However, their compressive strength remains inadequate for load-bearing applications, and rapid degradation often precedes complete tissue regeneration, necessitating covalent stabilization or composite reinforcement.

#### 6.2.1 Cartilage tissue repair

**FIGURE 6:**
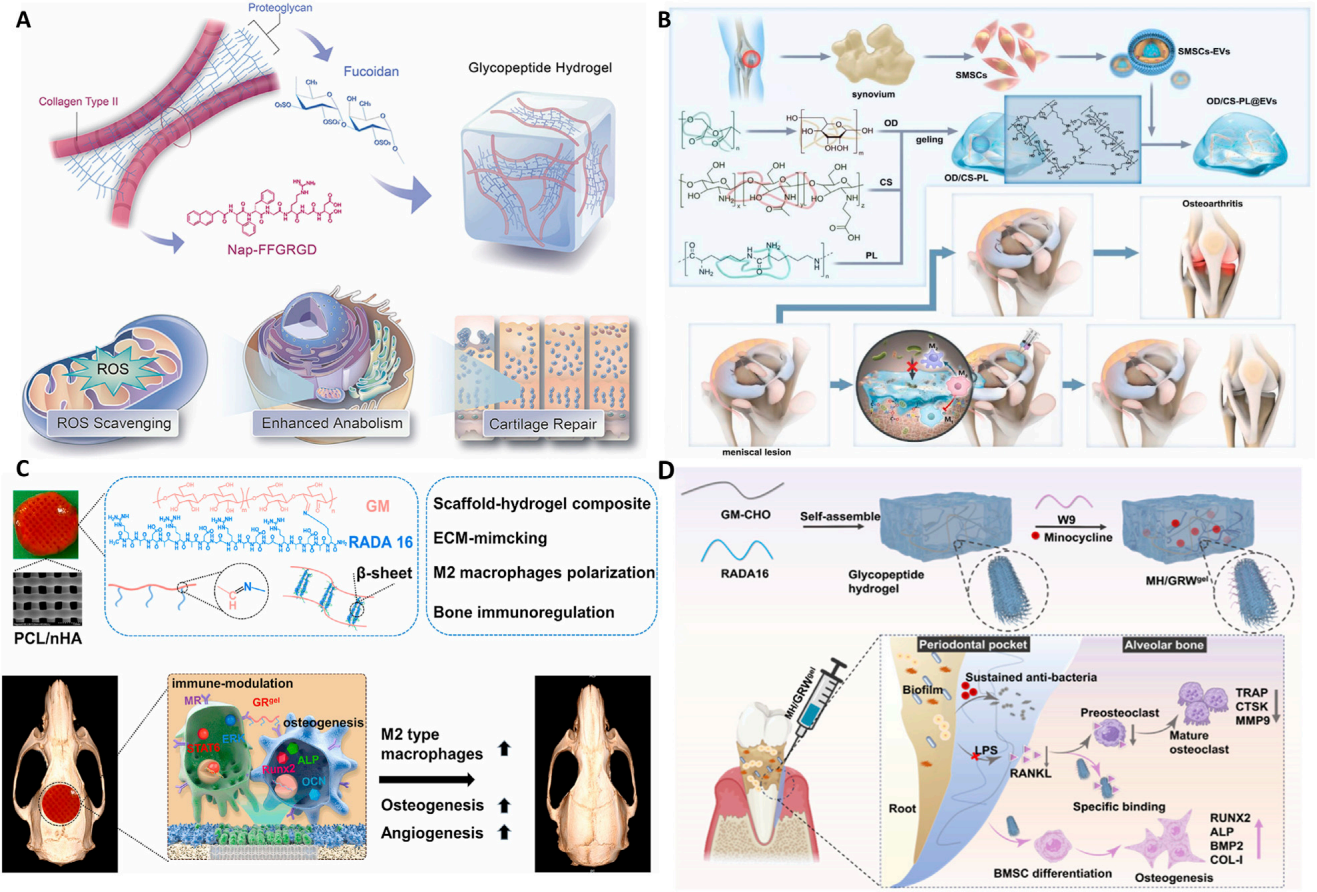
Application of glycopeptide hydrogels in bone defect repair. **(A)** Schematic diagram of the preparation and biological functions of the Nap-FFGRGD@Fucoidan hydrogel. (Zhao et al., 2024a), Copyright 2023. Reproduced with permission from Elsevier Science, Ltd. **(B)** Schematic diagram of the preparation and biological functions of the OD/CS-PL@EV hydrogel. (Huang et al., 2025), Copyright 2024. Reproduced with permission from Elsevier Science, Ltd. **(C)** Schematic diagram of the preparation and biological functions of PH@GR(gel). (Wang et al., 2022), Copyright 2022. Reproduced with permission from Elsevier Science, Ltd. **(D)** Schematic diagram of MH/GRW(gel). (Zhao et al., 2024b), Copyright 2024. Reproduced with permission from the American Chemical Society. CS: chitosan; EV: Extracellular vesicle; GRW: glucomannan, RADA16 and WP9QY peptide; PL: ε-Poly-L-lysine; OD: oxidized dextran; MH: minocycline hydrochloride; PH@GR(gel): glycopeptide hydrogel (GRgel) combined with a 3D-printed polycaprolactone and nano-hydroxyapatite (PCL/nHA) scaffold.

Cartilage is extremely difficult to repair due to its lack of a blood supply and low cellular activity; furthermore, cartilage damage is often accompanied by oxidative stress imbalances, ECM degradation, and deterioration of the inflammatory microenvironment (Jeyaraman et al., 2024). To address these challenges, researchers have been developing biomimetic scaffold materials that promote cartilage regeneration. The choice of scaffold material is critical for successfully reconstructing cartilage tissue, as the materials must have the appropriate biocompatibility, functionality, and mechanical properties. Zhao et al. (Zhao et al., 2024a) developed a biomimetic fibrous network glycopeptide hydrogel (Nap-FFGRGD@Fucoidan) based on marine-derived fucoidan and Nap-FFGRGD (Figure 6A), which mimicked the collagen fiber structure of natural cartilage. The hydrogel effectively eliminated ROS and increased the chondrocyte ECM synthesis capacity through the antioxidant action of fucoidan and cell adhesion sites of the RGD sequence, re-establishing the metabolic balance of the cartilage matrix. The study showed that the hydrogel significantly enhanced cartilage regeneration by activating the NRF2 antioxidant signaling pathway. *In vivo* experiments demonstrated that the hydrogel promoted new cartilage formation, increased the area of regenerated cartilage by 1.65 times, and coordinated subchondral bone structure remodeling, thus providing a novel approach to cartilage tissue engineering.

Hu et al. (2023) developed a novel glycopeptide hydrogel designed as a proteoglycan mimic for repairing articular cartilage defects. Using a Schiff base reaction, PLL was crosslinked with oxidized dextran to fabricate six hydrogel variants (Gel-1 to Gel-6) with gradient concentrations and distinct pore sizes. Among these, Gel-3—characterized by a pore size of 122 ± 12 μm—demonstrated superior cartilage regeneration capacity. The hydrogel had a loose porous structure that promoted chondrocyte proliferation, attachment, and migration, and significantly reduced intracellular ROS. *In vitro* and *in vivo* experiments demonstrated that the hydrogel promoted cartilage ECM deposition and upregulated cartilage-specific gene expression. In a rabbit knee cartilage defect model, the Gel-3 group exhibited excellent cartilage regeneration potential, thereby providing a theoretical reference for designing future cartilage tissue regeneration materials.

#### 6.2.2 Meniscus injury

The meniscus is an avascular, heterogeneous fibrocartilage structure with a low cell density that functions in knee joint stability and load distribution. However, its ability to regenerate and heal after tearing is limited (Yan W. et al., 2021). Current treatment methods, such as meniscus suturing, replacement, and tissue engineering, are challenged by biocompatibility, mechanical properties, and low healing rates. Huang et al. (Huang et al., 2025) designed and prepared a high-moisture adhesive antibacterial hydrogel (OD/CS-PL@EVs) via Schiff base and amide reactions to crosslink oxidized dextran, chitosan, and the antimicrobial peptide EPL, and formed a stable 3D network structure. The hydrogel was loaded with EVs derived from synovial mesenchymal stem cells (SMSCs) to enhance the bioactivity and repair function of the hydrogel (Figure 6B). When the hydrogel was injected to enable adhesive repair of meniscus injuries, it effectively adhered to the damaged tissue despite the moist joint environment and prevented microbial invasion. The SMSC-EVs loaded into the hydrogel promoted the proliferation, migration, and chondrogenic differentiation of endogenous cells. The hydrogel accelerated meniscus cartilage tissue regeneration and restored cartilage-like tissue and physiological function in the avascular region. Compared with current approaches for meniscus injuries, the hydrogel provided significant advantages in tissue integration, inflammation regulation, immune adaptability, and ease of operation, rendering it suitable for *in situ* repair of meniscus tears and for use in combination with meniscus suturing techniques for larger area injuries.

#### 6.2.3 Cranial and periodontal bone repair

To resolve large cranial bone defects, Wang et al. (Wang et al., 2022) designed a biomimetic glycopeptide hydrogel (GRgel) combined with a 3D-printed polycaprolactone and nano-hydroxyapatite (PCL/nHA) scaffold to form the PH@GRgel. The β-sheet RADA16 peptide was grafted onto glucomannan in the hydrogel to form an ECM-like fibrous structure as a non-covalent composite with a 3D-printed PCL/nHA scaffold. The hydrogel significantly promoted the proliferation and osteogenic differentiation of bone marrow mesenchymal stem cells (BMSCs) and induced M2 macrophage polarization, which enhanced macrophage-BMSC signaling communication (Figure 6C). In a rat cranial defect model, the composite scaffold exhibited 83.3% new bone formation with high levels of vascularization at the defect site after 12 weeks, demonstrating its immense potential for cranial regeneration.

To treat periodontal bone defects, Zhao et al. (Zhao et al., 2024b) designed a glycopeptide hydrogel (MH/GRW(gel)) loaded with MH. The hydrogel’s ECM-like fibrous and porous structure provided a scaffold supporting cell adhesion and proliferation. The hydrogel rapidly released MH to combat bacteria and effectively inhibited biofilm formation in deep periodontal pockets. More importantly, W9 specifically bound RANKL, blocking the RANKL/RANK signaling axis, inhibiting osteoclast maturation, and promoting osteoblastic differentiation. This significantly inhibited periodontal bone resorption and enhanced new bone formation in a rat periodontitis model, providing an innovative drug-free delivery solution for periodontal regeneration (Figure 6D).

### 6.3 Glycopeptide hydrogels in myocardial tissue repair

The treatment of myocardial infarction remains a significant challenge due to excessive inflammation, massive cell death, and limited regenerative potential, which leads to maladaptive healing processes and eventual heart failure. Ideal cardiac tissue engineering materials should both improve the delivery and function of cells and molecules and provide mechanical support to damaged and weakened cardiac tissue (Ye and Black, 2011; Chen and Liu, 2016). The current means of modulating inflammation and improving cardiac tissue regeneration have achieved only limited success. Furthermore, ideal cardiac regenerative biomaterials would be biomimetic and provide biological, mechanical, electrical, and chemical cues similar to those in the native myocardium, while being deliverable via minimally invasive procedures to reduce additional damage incurred during surgical implantation.

**FIGURE 7:**
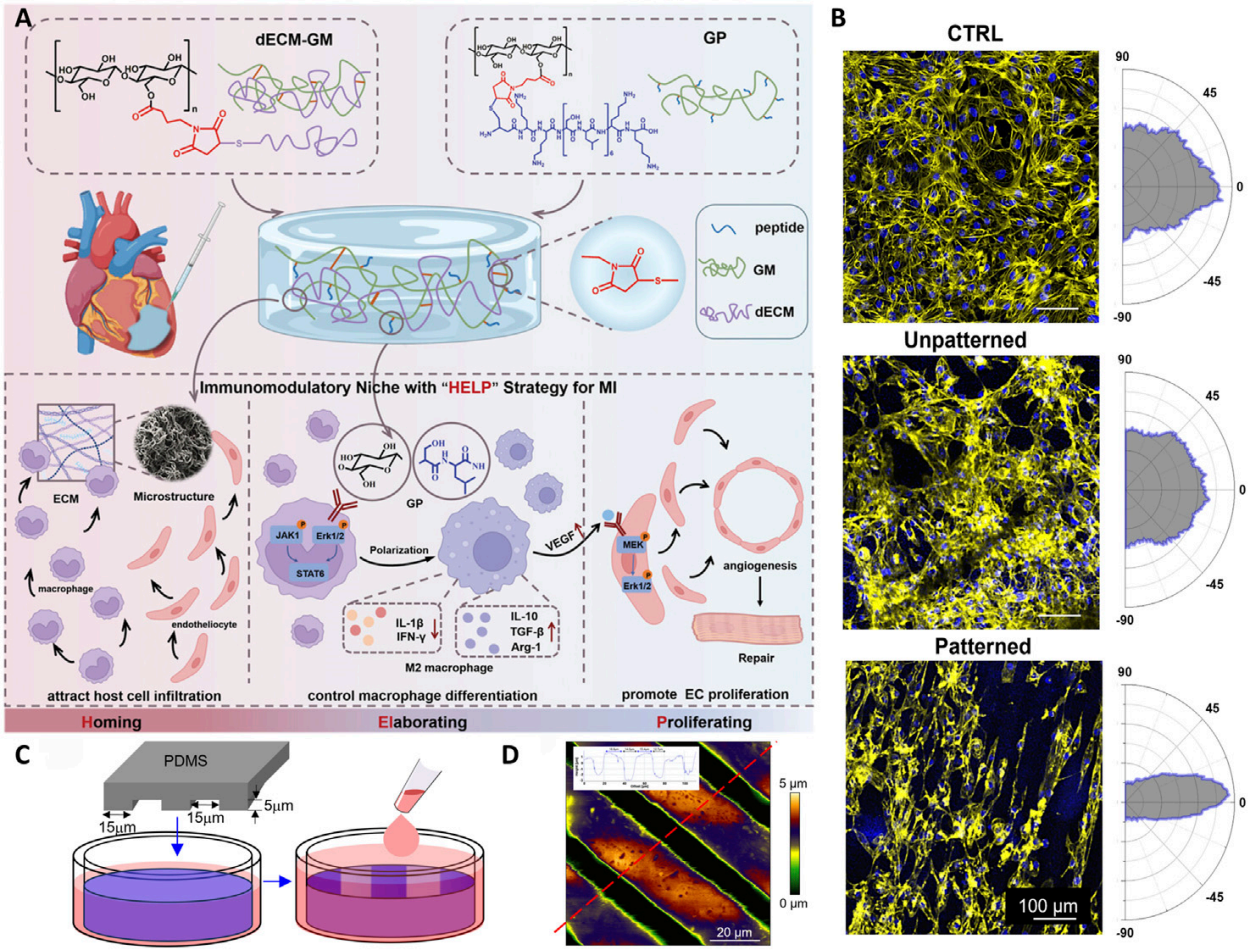
Application of glycopeptide hydrogels in myocardial tissue repair. **(A)** Schematic representation of the preparation and biological functions of dECM/GP. (Kong et al., 2023), Copyright 2023. Reproduced with permission from John Wiley and Sons, Inc. **(B)** Illustration of the elongation and alignment of iPSC-CMs cultured on control and Fmoc-FFGlcN6S hydrogel matrices. **(C)** Schematic of the micro-patterning process used. **(D)** Representative atomic force microscopy images of the micro-patterned hydrogels. (Castro et al., 2025), Copyright 2024. Reproduced with permission from Elsevier. dECM: decellularised extracellular matrix; GP: glycopeptide; iPSC-CMs: Induced pluripotent stem cell-derived cardiomyocytes.

In light of this, Kong et al. (Kong et al., 2023) developed a hybrid dECM/GP hydrogel comprising cardiac dECM and immunomodulatory glycopeptides to achieve endogenous tissue regeneration after myocardial infarction. The hydrogel mimicked the natural ECM structure to direct host cell homing, controlled macrophage differentiation through glycopeptide units, and promoted endothelial cell proliferation by enhancing macrophage-endothelial cell crosstalk, thereby coordinating the innate healing mechanisms of cardiac tissue regeneration (Figure 7A). In rodent myocardial infarction models, the hybrid hydrogel demonstrated enhanced M2 macrophage polarization, increased angiogenesis, and improved cardiomyocyte survival, effectively reducing the infarct size, improving wall thickness, and enhancing myocardial contractility.

Castro et al. (Castro et al., 2025) developed a dynamic supramolecular hydrogel (Fmoc-FFGlcN6S) based on the conjugation of diphenylalanine glucosamine-6-sulfate with fluorenylmethoxycarbonyl (Fmoc), which self-assembled into a nanofiber network simulating cardiac tissue. The hydrogel surface was micro-patterned to form grooves 15 μm wide (Figures 7C,D). This pattern guided the directional alignment of pluripotent stem cell-derived cardiomyocytes (iPSC-CMs) upon induction (Figure 7B). The cultured cardiomyocytes exhibited synchronous contractions, electrical interconnections, and mature marker expression, indicating their potential for providing critical physical and biochemical signals to facilitate myocardial tissue regeneration. The directional alignment promoted cardiomyocyte functionalization, enabling the cells to mimic the natural contraction patterns in cardiac tissue. This study provided new insights into cardiac tissue engineering.

### 6.4 Glycopeptide hydrogel applications in ocular treatment

(Xu et al., 2012) developed a therapeutic glycopeptide based on small peptide molecules, in which glucosamine groups were covalently attached to the peptide sequence (FMOC-Phe-Phe-Asp). The glycopeptide self-assembled into a hydrogel with a nanofiber microstructure under physiological conditions. When used in rabbit ocular filtration surgery, the hydrogel effectively inhibited the formation of postoperative scar tissue, maintained the patency of the postoperative filtering bleb and drainage fistula, and thus maintained a low intraocular pressure level for 21 days. The therapeutic effect was comparable to traditional anti-proliferative drugs, but without the need for additional anti-proliferative agents, thereby avoiding the toxic side effects of these drugs on ocular tissues. This also simplified the surgical procedure and reduced treatment costs, providing a safe and efficient alternative for glaucoma filtration surgery. However, precise control over degradation rates remains challenging in the enzymatically active ocular environment, and residual crosslinkers may provoke chronic inflammation unless rigorously purified.

### 6.5 Glycopeptide hydrogel applications in nerve repair

Damage to the central nervous system often causes neuronal loss, axonal rupture, and inflammation. The ECM, as a complex dynamic network, provides mechanical support to cells and regulates neuronal survival, proliferation, and differentiation through biochemical signals. Therefore, developing biomimetic hydrogels with ECM-like structures has become an important approach for promoting nerve repair (Raspa and Gelain, 2021). Despite progress, mismatched mechanical properties and asynchronous degradation with axonal regrowth limit functional recovery.

**FIGURE 8:**
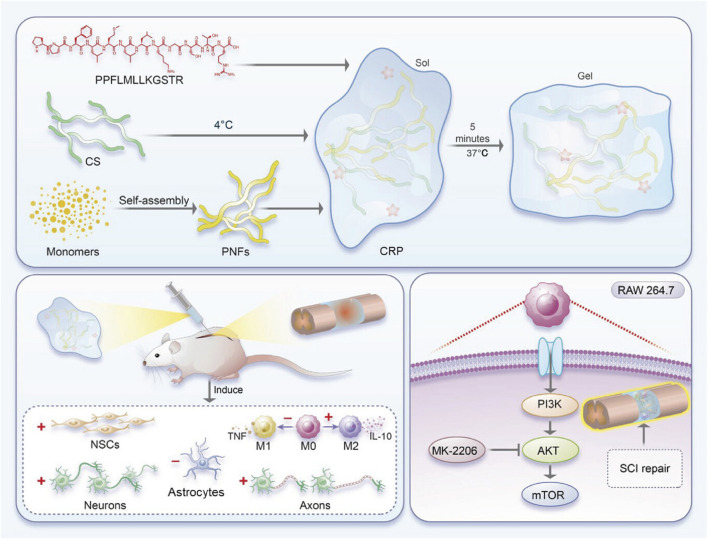
Preparation of the CRP hydrogel and its application in spinal cord injury. (Sun et al., 2024), Copyright 2024. Reproduced with permission from John Wiley and Sons, Inc. CRP: chitosan, RADA16 and PPFLMLLKGSTR peptide.

Glycopeptide hydrogels self-assemble to form highly hydrated nanofiber networks that effectively mimic the structure and function of the ECM. For example, Sun et al. (Sun et al., 2024) designed a composite CRP hydrogel,which rapidly gelled at body temperature and provided an ideal regenerative microenvironment for neural stem cells (NSCs) with its porous 3D structure. *In vitro* experiments showed that the CRP hydrogel promoted NSC proliferation and migration and significantly induced NSC differentiation into neurons while inhibiting their differentiation into glial cells. *In vivo* tests further validated the repair capacity of the CRP hydrogel. It improved motor function in a complete spinal cord injury model by regulating the PI3K/AKT/mTOR signaling pathway, reducing inflammation and glial scar formation (Figure 8). Castro et al. (Castro et al., 2023) developed a supramolecular hydrogel based on aromatic glycopeptides that captured and protected key growth factors such as FGF-2. The hydrogel-induced human adipose-derived stem cells to differentiate into the neural lineage without exogenous differentiation factors. The hydrogel significantly upregulated the expression of neural markers, such as GFAP and Nestin. Its mechanical properties were modulated to influence cell adhesion and differentiation efficiency through the degree of glycosylation. The level of glycosylation crucially affected the biological functionality of the hydrogel, conferring with excellent cell compatibility and neural differentiation effects.

## 7 Summary and outlook

Glycopeptide hydrogels, as a new type of multifunctional biomaterial, have broad prospects in tissue repair, drug delivery, and antimicrobial therapy applications due to their ECM biomimetic structures, dynamic responsiveness, and high bioactivity. Glycopeptide hydrogels exhibit unique advantages: their sugar chains mimic the glycosaminoglycan functions of the natural ECM, directly participating in cell-matrix interactions, while peptide chains provide specific biological signals to guide cell migration and differentiation. In contrast to pure protein-based hydrogels, the dynamic crosslinked networks of glycopeptide systems allow easier mechanical property regulation while integrating the immunomodulatory properties of polysaccharides with the targeting functions of peptides. This combination demonstrates a distinctive “chemical-biological” dual-active synergistic effect. In recent years, researchers have made significant progress in design strategies, biofunctional optimization, and the application expansion of glycopeptide hydrogels. For example, glycopeptide hydrogels constructed through dynamic covalent bonds, such as Schiff base bonds and borate ester bonds, and supramolecular interactions such as hydrogen bonds, π–π stacking, and electrostatic interactions, exhibit excellent biocompatibility and degradability while supporting the regulation of mechanical properties, drug release, and immune microenvironment modulation according to specific environmental needs. In addition, researchers have successfully developed high-performance glycopeptide hydrogels that promote angiogenesis, regulate inflammatory responses, and enhance antibacterial activity by combining self-assembling peptides with functionalized polysaccharides, thereby providing innovative solutions for treating chronic wounds, cartilage injuries, and other complex diseases.

The future development of glycopeptide hydrogels should focus on systematic exploration across three dimensions: functional module expansion, biomechanical mimicry, and organ microenvironment adaptation, driving their evolution from “structural support” to “functional regeneration” materials. First, the expansion and precision design of functional peptide motifs will be pivotal for enhancing hydrogel bioactivity. While classical sequences such as RGD have been widely employed to promote cell adhesion and migration, their regulatory capacity in specific tissue microenvironments remains limited. Future research could integrate structural prediction technologies (e.g., AlphaFold-Multimer) with proteomics data to screen or design peptide analogs with multifunctional capabilities, such as neural guidance, angiogenesis, and immunomodulation, thereby constructing a synergistic glycopeptide hydrogel platform. Additionally, leveraging the inherent modifiability of polysaccharide chains, glycosylation modulation strategies (e.g., oligosaccharide modification, site-specific polysaccharide functionalization) could further enhance peptide recognition and signaling efficiency in complex microenvironments, broadening the functional scope of these materials.

Second, the current glycopeptide hydrogels struggle to match the complex mechanical demands of natural soft tissues in terms of toughness, shear recovery, and stress relaxation. Enhancing viscoelastic properties is a core challenge for achieving tissue biomimicry and dynamic adaptability. Building on recent advances in high-strength hydrogels, future glycopeptide systems could incorporate dual-network architectures, slide-ring structure, and polymer chain entanglement strategies to establish multi-scale hierarchical mechanical networks. These networks would enable energy dissipation and structural stability under high-strain conditions. Furthermore, integrating elastin-mimetic sequences, photo-responsive dynamic crosslinking, and reversible non-covalent “sacrificial bonds” may further refine the hydrogel’s viscoelastic adaptability (Teng et al., 2025).

Finally, bio-inspired designs tailored to multi-organ microenvironments will expand the applications of glycopeptide hydrogels in regenerative medicine. Given the unique ECM composition, structural hierarchy, and mechanical properties of different tissues and organs, universal hydrogels are inadequate for precise repair. Future efforts should leverage spatial transcriptomics and single-cell ECM omics data to decode organ-specific matrix compositions and glycosylation patterns, guiding the microenvironment-mimetic design of glycopeptide hydrogels.

In summary, as a new generation of functionally integrated biomaterials, the advancement of glycopeptide hydrogels requires coordinated progress across three critical pathways: functional sequence expansion, dynamic mechanical regulation, and tissue microenvironment adaptation. This will facilitate their transition from “ECM mimicry” to “regenerative regulation,” laying a robust foundation for their broad application in precision medicine and personalized tissue engineering.

## Abbreviations

3D, Three-dimensional; AMP, Antimicrobial peptide; AST, Astragaloside IV; BSP, Bletilla striata polysaccharide; dECM, Decellularised extracellular matrix; ECM, Extracellular matrix; EGCG, Epigallocatechin-3-gallate; EPL, ε-Poly-L-lysine; EVs, Extracellular vesicles; FF, Diphenylalanine; Fmoc, Fluorenylmethoxycarbonyl; GA, Gallic acid; GM, Glucomannan; HA, Hyaluronic acid; MH, Minocycline hydrochloride; Nap, Naphthalene; PAP, Pro-angiogenic peptide; PBA, Phenylboronic acid; PEG, Polyethylene glycol; PLL, Poly-L-lysine; RADA16, RADARADARADARADA; ROS, Reactive oxygen species; TBA, 2,3,4-Trihydroxybenzaldehyde; TGF-β, Transforming growth factor-β; TNF-α, Tumor necrosis factor-α; W9, WP9QY.

## References

[B1] Aoki K. Saito H. Itzstein C. Ishiguro M. Shibata T. Blanque R. (2006). A TNF receptor loop peptide mimic blocks RANK ligand–induced signaling, bone resorption, and bone loss. J. Clin. Invest 116 (6), 1525–1534. 10.1172/jci22513 16680194 PMC1448165

[B2] Apostolova E. Lukova P. Baldzhieva A. Katsarov P. Nikolova M. Iliev I. (2020). Immunomodulatory and anti-inflammatory effects of fucoidan: a review. Polymers 12 (10), 2338. 10.3390/polym12102338 33066186 PMC7602053

[B3] Azuri I. Adler-Abramovich L. Gazit E. Hod O. Kronik L. (2014). Why are diphenylalanine-based peptide nanostructures so rigid? Insights from first principles calculations. J. Am. Chem. Soc. 136 (3), 963–969. 10.1021/ja408713x 24368025

[B4] Bai L. Wang T. Deng Q. Zheng W. Li X. Yang H. (2024). Dual properties of pharmacological activities and preparation excipient: Bletilla striata polysaccharides. Int. J. Biol. Macromol. 254, 127643. 10.1016/j.ijbiomac.2023.127643 37898246

[B5] Baranwal A. Kumar A. Priyadharshini A. Oggu G. S. Bhatnagar I. Srivastava A. (2018). Chitosan: an undisputed bio-fabrication material for tissue engineering and bio-sensing applications. Int. J. Biol. Macromol. 110, 110–123. 10.1016/j.ijbiomac.2018.01.006 29339286

[B6] Bernardi A. Jiménez-Barbero J. Casnati A. De Castro C. Darbre T. Fieschi F. (2013). Multivalent glycoconjugates as anti-pathogenic agents. Chem. Soc. Rev. 42 (11), 4709–4727. 10.1039/c2cs35408j 23254759 PMC4399576

[B7] Bertsch P. Diba M. Mooney D. J. Leeuwenburgh S. C. G. (2023). Self-healing injectable hydrogels for tissue regeneration. Chem. Rev. 123 (2), 834–873. 10.1021/acs.chemrev.2c00179 35930422 PMC9881015

[B8] Bhagat V. Becker M. L. (2017). Degradable adhesives for surgery and tissue engineering. Biomacromolecules 18 (10), 3009–3039. 10.1021/acs.biomac.7b00969 28862846

[B9] Bhattacharya D. S. Svechkarev D. Souchek J. Hill T. K. Taylor M. Natarajan A. (2017). Impact of structurally modifying hyaluronic acid on CD44 interaction. J. Mater. Chem. B 5 (41), 8183–8192. 10.1039/c7tb01895a 29354263 PMC5773055

[B10] Binaymotlagh R. Chronopoulou L. Haghighi F. H. Fratoddi I. Palocci C. (2022). Peptide-based hydrogels: new materials for biosensing and biomedical applications. Materials 15 (17), 5871. 10.3390/ma15175871 36079250 PMC9456777

[B11] Blacklow S. O. Li J. Freedman B. R. Zeidi M. Chen C. Mooney D. J. (2019). Bioinspired mechanically active adhesive dressings to accelerate wound closure. Sci. Adv. 5 (7), eaaw3963. 10.1126/sciadv.aaw3963 31355332 PMC6656537

[B12] Bloom S. I. Islam M. T. Lesniewski L. A. Donato A. J. (2023). Mechanisms and consequences of endothelial cell senescence. Nat. Rev. Cardiol. 20 (1), 38–51. 10.1038/s41569-022-00739-0 35853997 PMC10026597

[B13] Bouten P. J. M. Zonjee M. Bender J. Yauw S. T. K. Van Goor H. Van Hest J. C. M. (2014). The chemistry of tissue adhesive materials. Prog. Polym. Sci. 39 (7), 1375–1405. 10.1016/j.progpolymsci.2014.02.001

[B14] Brown B. N. Ratner B. D. Goodman S. B. Amar S. Badylak S. F. (2012). Macrophage polarization: an opportunity for improved outcomes in biomaterials and regenerative medicine. Biomaterials 33 (15), 3792–3802. 10.1016/j.biomaterials.2012.02.034 22386919 PMC3727238

[B15] Cambre J. N. Sumerlin B. S. (2011). Biomedical applications of boronic acid polymers. Polymer 52 (21), 4631–4643. 10.1016/j.polymer.2011.07.057

[B16] Castro V. I. B. Amorim S. Caballero D. Abreu C. M. Kundu S. C. Reis R. L. (2025). Patterned glycopeptide-based supramolecular hydrogel promotes the alignment and contractility of iPSC-derived cardiomyocytes. Biomater. Adv. 167, 214091. 10.1016/j.bioadv.2024.214091 39500148

[B17] Castro V. I. B. Araújo A. R. Duarte F. Sousa-Franco A. Reis R. L. Pashkuleva I. (2023). Glycopeptide-based supramolecular hydrogels induce differentiation of adipose stem cells into neural lineages. ACS Appl. Mater Interfaces 15 (25), 29998–30007. 10.1021/acsami.3c05309 37327399 PMC10316323

[B18] Cecioni S. Imberty A. Vidal S. (2015). Glycomimetics versus multivalent glycoconjugates for the design of high affinity lectin ligands. Chem. Rev. 115 (1), 525–561. 10.1021/cr500303t 25495138

[B19] Chaudhuri O. Cooper-White J. Janmey P. A. Mooney D. J. Shenoy V. B. (2020). Effects of extracellular matrix viscoelasticity on cellular behaviour. Nature 584 (7822), 535–546. 10.1038/s41586-020-2612-2 32848221 PMC7676152

[B20] Chaudhuri O. Gu L. Klumpers D. Darnell M. Bencherif S. A. Weaver J. C. (2016). Hydrogels with tunable stress relaxation regulate stem cell fate and activity. Nat. Mater 15 (3), 326–334. 10.1038/nmat4489 26618884 PMC4767627

[B21] Chen F. M. Liu X. (2016). Advancing biomaterials of human origin for tissue engineering. Prog. Polym. Sci. 53, 86–168. 10.1016/j.progpolymsci.2015.02.004 27022202 PMC4808059

[B22] Chen P. Pan K. Song N. Yang Y. Gu C. Zhong P. (2023). A natural extracellular matrix hydrogel through selective nutrient restriction for hyperinflammatory starvation therapy. Matter 6 (2), 1037–1038. 10.1016/j.matt.2023.01.013

[B23] Chopra H. Kumar S. Singh I. (2022). Strategies and therapies for wound healing: a review. CDT 23 (1), 87–98. 10.2174/1389450122666210415101218 33858310

[B24] Cromwell O. R. Chung J. Guan Z. (2015). Malleable and self-healing covalent polymer networks through tunable dynamic boronic ester bonds. J. Am. Chem. Soc. 137 (20), 6492–6495. 10.1021/jacs.5b03551 25945818

[B25] Cui C. Liu W. (2021). Recent advances in wet adhesives: adhesion mechanism, design principle and applications. Prog. Polym. Sci. 116, 101388. 10.1016/j.progpolymsci.2021.101388

[B26] Degtyar E. Harrington M. J. Politi Y. Fratzl P. (2014). The mechanical role of metal ions in biogenic protein‐based materials. Angew. Chem. Int. Ed. 53 (45), 12026–12044. 10.1002/anie.201404272 25303013

[B27] Del Gaudio P. Amante C. Civale R. Bizzarro V. Petrella A. Pepe G. (2020). *In situ* gelling alginate-pectin blend particles loaded with Ac2-26: a new weapon to improve wound care armamentarium. Carbohydr. Polym. 227, 115305. 10.1016/j.carbpol.2019.115305 31590879

[B28] Deng X. Wu Y. Tang Y. Ge Z. Wang D. Zheng C. (2024). Microenvironment-responsive smart hydrogels with antibacterial activity and immune regulation for accelerating chronic wound healing. J. Control Release 368, 518–532. 10.1016/j.jconrel.2024.03.002 38462042

[B29] Díaz-Montes E. (2021). Dextran: sources, structures, and properties. Polysaccharides 2 (3), 554–565. 10.3390/polysaccharides2030033

[B30] Dicker K. T. Gurski L. A. Pradhan-Bhatt S. Witt R. L. Farach-Carson M. C. Jia X. (2014). Hyaluronan: a simple polysaccharide with diverse biological functions. Acta biomater. 10 (4), 1558–1570. 10.1016/j.actbio.2013.12.019 24361428 PMC3960342

[B31] Dodda J. M. Tsai S.-W. Ashammakhi N. (2024). Injectable Smart Hydrogels: Introduction, Preparation, and Applications, Royal Society of Chemistry. 17 (1) 1–27. 10.1039/BK9781837673070-00001

[B32] Faheem S. Hameed H. Paiva-Santos A. C. Khan M. A. Ghumman S. A. Hameed A. (2024). The role of chondroitin sulphate as a potential biomaterial for hepatic tissue regeneration: a comprehensive review. Int. J. Biol. Macromol. 280, 136332. 10.1016/j.ijbiomac.2024.136332 39482129

[B33] Fahmy‐Garcia S. Mumcuoglu D. De Miguel L. Dieleman V. Witte‐Bouma J. Van Der Eerden B. C. J. (2018). Novel *in situ* gelling hydrogels loaded with recombinant collagen peptide microspheres as a slow‐release system induce ectopic bone formation. Adv. Healthc. Mater. 7 (21), 1800507. 10.1002/adhm.201800507 30230271

[B34] Fan Z. Cheng P. Yin G. Wang Z. Han J. (2020). *In situ* forming oxidized salecan/gelatin injectable hydrogels for vancomycin delivery and 3D cell culture. J. Biomaterials Sci. Polym. Ed. 31 (6), 762–780. 10.1080/09205063.2020.1717739 31944896

[B35] Feng Z. Su Q. Zhang C. Huang P. Song H. Dong A. (2020). Bioinspired nanofibrous glycopeptide hydrogel dressing for accelerating wound healing: a cytokine-free, M2-type macrophage polarization approach. Adv. Funct. Mater 30 (52), 2006454. 10.1002/adfm.202006454

[B36] Feng Z. Zhang Y. Yang C. Liu X. Huangfu Y. Zhang C. (2023). Bioinspired and inflammation-modulatory glycopeptide hydrogels for radiation-induced chronic skin injury repair. Adv. Healthc. Mater 12 (1), e2201671. 10.1002/adhm.202201671 36183357

[B37] Galler K. M. Aulisa L. Regan K. R. D’Souza R. N. Hartgerink J. D. (2010). Self-assembling multidomain peptide hydrogels: designed susceptibility to enzymatic cleavage allows enhanced cell migration and spreading. J. Am. Chem. Soc. 132 (9), 3217–3223. 10.1021/ja910481t 20158218 PMC2857986

[B38] Geng X. Wang Y. Cui H. Li C. Cheng B. Cui B. (2023). Carboxymethyl chitosan regulates macrophages polarization to inhibit early subconjunctival inflammation in conjunctival injury. Int. J. Biol. Macromol. 244, 125159. 10.1016/j.ijbiomac.2023.125159 37268068

[B39] George M. Abraham T. E. (2006). Polyionic hydrocolloids for the intestinal delivery of protein drugs: alginate and chitosan — a review. J. Control. Release 114 (1), 1–14. 10.1016/j.jconrel.2006.04.017 16828914

[B40] Ghattas M. Dwivedi G. Chevrier A. Horn-Bourque D. Alameh M. G. Lavertu M. (2025). Chitosan immunomodulation: insights into mechanisms of action on immune cells and signaling pathways. RSC Adv. 15 (2), 896–909. 10.1039/d4ra08406c 39802469 PMC11719903

[B41] Gou K. Li Y. Qu Y. Li H. Zeng R. (2022). Advances and prospects of Bletilla striata polysaccharide as promising multifunctional biomedical materials. Mater. and Des. 223, 111198. 10.1016/j.matdes.2022.111198

[B42] Guan T. Li J. Chen C. Liu Y. (2022). Self‐assembling peptide‐based hydrogels for wound tissue repair. Adv. Sci. 9 (10), 2104165. 10.1002/advs.202104165 PMC8981472 35142093

[B43] Guo J. Zhang X. Mao R. Li H. Hao Y. Zhang J. (2024). Multifunctional glycopeptide-based hydrogel via dual-modulation for the prevention and repair of radiation-induced skin injury. ACS Biomater. Sci. Eng. 10 (8), 5168–5180. 10.1021/acsbiomaterials.4c00698 39016069

[B44] Guo S. DiPietro L. A. (2010). Factors affecting wound healing. J. Dent. Res. 89 (3), 219–229. 10.1177/0022034509359125 20139336 PMC2903966

[B45] Han Y. Cao Y. Lei H. (2022). Dynamic covalent hydrogels: strong yet dynamic. Gels 8 (9), 577. 10.3390/gels8090577 36135289 PMC9498565

[B46] Hao Y. Li H. Guo J. Wang D. Zhang J. Liu J. (2023). Bio‐inspired antioxidant heparin‐mimetic peptide hydrogel for radiation‐induced skin injury repair. Adv. Healthc. Mater. 12 (20), 2203387. 10.1002/adhm.202203387 36934301

[B47] Hofman A. H. Van Hees I. A. Yang J. Kamperman M. (2018). Bioinspired underwater adhesives by using the supramolecular toolbox. Adv. Mater. 30 (19), 1704640. 10.1002/adma.201704640 29356146

[B48] Holten-Andersen N. Jaishankar A. Harrington M. J. Fullenkamp D. E. DiMarco G. He L. (2014). Metal-coordination: using one of nature’s tricks to control soft material mechanics. J. Mater Chem. B 2 (17), 2467–2472. 10.1039/c3tb21374a 26413297 PMC4582448

[B49] Hu Y. Lyu C. Teng L. Wu A. Zhu Z. He Y. (2023). Glycopolypeptide hydrogels with adjustable enzyme-triggered degradation: a novel proteoglycans analogue to repair articular-cartilage defects. Mater. Today Bio 20, 100659. 10.1016/j.mtbio.2023.100659 PMC10205498 37229212

[B50] Hua Y. Gan Y. Zhang Y. Ouyang B. Tu B. Zhang C. (2019). Adaptable to mechanically stable hydrogels based on the dynamic covalent cross-linking of thiol-aldehyde addition. ACS Macro Lett. 8 (3), 310–314. 10.1021/acsmacrolett.9b00020 35650834

[B51] Huang M. Yuan Z. Fu G. Dong J. Sun Y. Wang W. (2025). An injectable antibacterial wet-adhesive for meniscal cartilage regeneration via immune homeostasis mediated by SMSC-derived extracellular vesicles. Compos. Part B Eng. 291, 111970. 10.1016/j.compositesb.2024.111970

[B52] Huang R. Qi W. Feng L. Su R. He Z. (2011). Self-assembling peptide–polysaccharide hybrid hydrogel as a potential carrier for drug delivery. Soft Matter 7 (13), 6222. 10.1039/c1sm05375b

[B53] Hussain M. Javeed A. Ashraf M. Zhao Y. Mukhtar M. M. Rehman M. U. (2012). Aspirin and immune system. Int. Immunopharmacol. 12 (1), 10–20. 10.1016/j.intimp.2011.11.021 22172645

[B54] Hyldgaard M. Mygind T. Vad B. S. Stenvang M. Otzen D. E. Meyer R. L. (2014). The antimicrobial mechanism of action of epsilon-poly-l -lysine. Appl. Environ. Microbiol. 80 (24), 7758–7770. 10.1128/aem.02204-14 25304506 PMC4249222

[B55] Imberty A. Varrot A. (2008). Microbial recognition of human cell surface glycoconjugates. Curr. Opin. Struct. Biol. 18 (5), 567–576. 10.1016/j.sbi.2008.08.001 18809496

[B56] Jaipan P. Nguyen A. Narayan R. J. (2017). Gelatin-based hydrogels for biomedical applications. MRS Commun. 7 (3), 416–426. 2017/10/03 ed. 10.1557/mrc.2017.92

[B57] Jeyaraman M. Jeyaraman N. Nallakumarasamy A. Ramasubramanian S. Yadav S. (2024). Critical challenges and frontiers in cartilage tissue engineering. Cureus 16 (1), e53095. 10.7759/cureus.53095 38414693 PMC10897756

[B58] Jomova K. Raptova R. Alomar S. Y. Alwasel S. H. Nepovimova E. Kuca K. (2023). Reactive oxygen species, toxicity, oxidative stress, and antioxidants: chronic diseases and aging. Arch. Toxicol. 97 (10), 2499–2574. 10.1007/s00204-023-03562-9 37597078 PMC10475008

[B59] Jung J. P. Nagaraj A. K. Fox E. K. Rudra J. S. Devgun J. M. Collier J. H. (2009). Co-assembling peptides as defined matrices for endothelial cells. Biomaterials 30 (12), 2400–2410. 10.1016/j.biomaterials.2009.01.033 19203790 PMC2677558

[B60] Kim J. Park W. Min B. (2005). The PPFLMLLKGSTR motif in globular domain 3 of the human laminin-5 α3 chain is crucial for integrin α3β1 binding and cell adhesion. Exp. Cell Res. 304 (1), 317–327. 10.1016/j.yexcr.2004.11.009 15707596

[B61] Kong P. Dong J. Li W. Li Z. Gao R. Liu X. (2023). Extracellular matrix/glycopeptide hybrid hydrogel as an immunomodulatory niche for endogenous cardiac repair after myocardial infarction. Adv. Sci. 10 (23), 2301244. 10.1002/advs.202301244 PMC10427380 37318159

[B62] Kumar V. A. Taylor N. L. Shi S. Wang B. K. Jalan A. A. Kang M. K. (2015). Highly angiogenic peptide nanofibers. ACS Nano 9 (1), 860–868. 10.1021/nn506544b 25584521 PMC4370274

[B63] Lavrador P. Gaspar V. M. Mano J. F. (2020). Mechanochemical patternable ECM‐mimetic hydrogels for programmed cell orientation. Adv. Healthc. Mater. 9 (10), 1901860. 10.1002/adhm.201901860 32323469

[B64] Lee H. Y. Hwang C. H. Kim H. E. Jeong S. H. (2018). Enhancement of bio-stability and mechanical properties of hyaluronic acid hydrogels by tannic acid treatment. Carbohydr. Polym. 186, 290–298. 10.1016/j.carbpol.2018.01.056 29455990

[B65] Li J. Jiang X. Li H. Gelinsky M. Gu Z. (2021a). Tailoring materials for modulation of macrophage fate. Adv. Mater. 33 (12), 2004172. 10.1002/adma.202004172 PMC9245340 33565154

[B66] Li J. Liang S. Yan Y. Tian X. Li X. (2019a). O-mannosylation affords a glycopeptide hydrogel with inherent antibacterial activities against *E. coli* via multivalent interactions between lectins and supramolecular assemblies. Macromol. Biosci. 19 (9), 1900124. 10.1002/mabi.201900124 31310440

[B67] Li L. Xiao B. Mu J. Zhang Y. Zhang C. Cao H. (2019b). A MnO_2_ nanoparticle-dotted hydrogel promotes spinal cord repair *via* regulating reactive oxygen species microenvironment and synergizing with mesenchymal stem cells. ACS Nano 13 (12), 14283–14293. 10.1021/acsnano.9b07598 31769966

[B68] Li Y. Wang J. Qian D. Chen L. Mo X. Wang L. (2021b). Electrospun fibrous sponge via short fiber for mimicking 3D ECM. J. Nanobiotechnol 19 (1), 131. 10.1186/s12951-021-00878-5 PMC8106196 33964948

[B69] Li Z. Lu J. Ji T. Xue Y. Zhao L. Zhao K. (2024). Self‐healing hydrogel bioelectronics. Adv. Mater. 36 (21), 2306350. 10.1002/adma.202306350 37987498

[B70] Liang Y. Chen B. Liang D. Quan X. Gu R. Meng Z. (2023). Pharmacological effects of astragaloside IV: a review. Molecules 28 (16), 6118. 10.3390/molecules28166118 37630371 PMC10458270

[B71] Lienhard G. E. Jencks W. P. (1966). Thiol addition to the carbonyl group. Equilibria and Kinetics^1^ . J. Am. Chem. Soc. 88 (17), 3982–3995. 10.1021/ja00969a017 5915153

[B72] Liu J. Sun Z. Yuan Y. Tian X. Liu X. Duan G. (2016). Peptide glycosylation generates supramolecular assemblies from glycopeptides as biomimetic scaffolds for cell adhesion and proliferation. ACS Appl. Mater Interfaces 8 (11), 6917–6924. 10.1021/acsami.6b00850 26930123

[B73] Liu S. Li H. Zhang J. Tian X. Li X. (2020). A biocompatible supramolecular hydrogel with multivalent galactose ligands inhibiting*Pseudomonas aeruginosa*virulence and growth. RSC Adv. 10 (56), 33642–33650. 10.1039/d0ra06718k 35519035 PMC9056750

[B74] Liu W. Gao R. Yang C. Feng Z. Ou-Yang W. Pan X. (2022). ECM-mimetic immunomodulatory hydrogel for methicillin-resistant *Staphylococcus aureus*-infected chronic skin wound healing. Sci. Adv. 8 (27), eabn7006. 10.1126/sciadv.abn7006 35857459 PMC9269894

[B75] Liu W. Liu S. Sun M. Guo F. Wang P. Jia L. (2024). Glycopeptide-based multifunctional nanofibrous hydrogel that facilitates the healing of diabetic wounds infected with methicillin-resistant *Staphylococcus aureus* . Acta Biomater. 181, 161–175. 10.1016/j.actbio.2024.04.035 38679405

[B76] Lukova P. Kokova V. Baldzhieva A. Murdjeva M. Katsarov P. Delattre C. (2024). Alginate from ericaria crinita possesses antioxidant activity and attenuates systemic inflammation via downregulation of pro-inflammatory cytokines. Mar. Drugs 22 (11), 482. 10.3390/md22110482 39590762 PMC11595431

[B77] Luo Y. Diao H. Xia S. Dong L. Chen J. Zhang J. (2010). A physiologically active polysaccharide hydrogel promotes wound healing. J. Biomed. Mater. Res. Part A An Official J. Soc. Biomaterials, Jpn. Soc. Biomaterials, Aust. Soc. Biomaterials Korean Soc. Biomaterials 94 (1), 193–204. 10.1002/jbm.a.32711 20128009

[B78] Ma X. Huang X. Wang A. Sun T. Tai R. Li J. (2024). *In situ* injectable photo-crosslinking hydrogel with heterojunction nanoparticles for dual-channel synergistic disinfection and cutaneous regeneration in diabetic chronic wound healing. Nano Today 56, 102235. 10.1016/j.nantod.2024.102235

[B79] Ma Z. Bao G. Li J. (2021). Multifaceted design and emerging applications of tissue adhesives. Adv. Mater. 33 (24), 2007663. 10.1002/adma.202007663 33956371

[B80] Manzoor A. Dar A. H. Pandey V. K. Shams R. Khan S. Panesar P. S. (2022). Recent insights into polysaccharide-based hydrogels and their potential applications in food sector: a review. Int. J. Biol. Macromol. 213, 987–1006. 10.1016/j.ijbiomac.2022.06.044 35705126

[B81] Marinho A. Nunes C. Reis S. (2021). Hyaluronic acid: a key ingredient in the therapy of inflammation. Biomolecules 11 (10), 1518. 10.3390/biom11101518 34680150 PMC8533685

[B82] Marinval N. Morenc M. Labour M. Samotus A. Mzyk A. Ollivier V. (2018). Fucoidan/VEGF-based surface modification of decellularized pulmonary heart valve improves the antithrombotic and re-endothelialization potential of bioprostheses. Biomaterials 172, 14–29. 10.1016/j.biomaterials.2018.01.054 29715592

[B83] Matsumura K. Nakajima N. Sugai H. Hyon S. H. (2014). Self-degradation of tissue adhesive based on oxidized dextran and poly-l-lysine. Carbohydr. Polym. 113, 32–38. 10.1016/j.carbpol.2014.06.073 25256455

[B84] Miao H. Elkin M. Aingorn E. Ishai‐Michaeli R. Stein C. A. Vlodavsky I. (1999). Inhibition of heparanase activity and tumor metastasis by laminarin sulfate and synthetic phosphorothioate oligodeoxynucleotides. Int. J. Cancer 83 (3), 424–431. 10.1002/(sici)1097-0215(19991029)83:3<424::aid-ijc20>3.3.co;2-c 10495437

[B85] Miura Y. Hoshino Y. Seto H. (2016). Glycopolymer nanobiotechnology. Chem. Rev. 116 (4), 1673–1692. 10.1021/acs.chemrev.5b00247 26509280

[B86] Mo C. Xiang L. Chen Y. (2021). Advances in injectable and self‐healing polysaccharide hydrogel based on the Schiff base reaction. Macromol. Rapid Commun. 42 (10), 2100025. 10.1002/marc.202100025 33876841

[B87] Mo C. Zhang W. Zhu K. Du Y. Huang W. Wu Y. (2024). Advances in injectable hydrogels based on diverse gelation methods for biomedical imaging. Small Methods 8 (12), 2400076. 10.1002/smtd.202400076 38470225

[B88] Moon A. Y. Bailey E. J. Polanco J. A. Kurata W. E. Pierce L. M. (2023). Antibacterial efficacy of a chitosan-based hydrogel modified with epsilon-poly-l -lysine against *Pseudomonas aeruginosa* in a murine-infected burn wound model. Mil. Med. 188 (Suppl. ment_6), 52–60. 10.1093/milmed/usad013 37948238

[B89] Moon K. H. Pack M. Y. (1983). Cytotoxicity of cinnamic aldehyde on leukemia L1210 cells. Drug Chem. Toxicol. 6 (6), 521–535. 10.3109/01480548309017807 6653439

[B90] Moore A. N. Lopez Silva T. L. Carrejo N. C. Origel Marmolejo C. A. Li I. C. Hartgerink J. D. (2018). Nanofibrous peptide hydrogel elicits angiogenesis and neurogenesis without drugs, proteins, or cells. Biomaterials 161, 154–163. 10.1016/j.biomaterials.2018.01.033 29421552 PMC5837816

[B91] Moura L. I. Dias A. M. Carvalho E. de Sousa H. C. (2013). Recent advances on the development of wound dressings for diabetic foot ulcer treatment—a review. Acta biomater. 9 (7), 7093–7114. 10.1016/j.actbio.2013.03.033 23542233

[B92] MubarakAli D. LewisOscar F. Gopinath V. Alharbi N. S. Alharbi S. A. Thajuddin N. (2018). An inhibitory action of chitosan nanoparticles against pathogenic bacteria and fungi and their potential applications as biocompatible antioxidants. Microb. Pathog. 114, 323–327. 10.1016/j.micpath.2017.11.043 29229504

[B93] Muro P. Zhang L. Li S. Zhao Z. Jin T. Mao F. (2024). The emerging role of oxidative stress in inflammatory bowel disease. Front. Endocrinol. 15, 1390351. 10.3389/fendo.2024.1390351 PMC11284038 39076514

[B94] Nagai Y. Unsworth L. D. Koutsopoulos S. Zhang S. (2006). Slow release of molecules in self-assembling peptide nanofiber scaffold. J. Control. release 115 (1), 18–25. 10.1016/j.jconrel.2006.06.031 16962196

[B95] Nazeer N. Ahmed M. (2025). “Peptide-based hydrogels,” in Natural and synthetic hydrogels (Elsevier), 115–149.

[B96] Niu X. Yu J. Huang Q. Yu J. Yang Y. Song H. (2022). Immunoenhancement activity of Bletilla striata polysaccharide through MAPK and NF-κB signalling pathways *in vivo* and *in vitro* . Autoimmunity 55 (8), 650–660. 10.1080/08916934.2022.2103801 35892187

[B97] Omar J. Ponsford D. Dreiss C. A. Lee T. Loh X. J. (2022). Supramolecular hydrogels: design strategies and contemporary biomedical applications. Chem. An Asian J. 17 (9), e202200081. 10.1002/asia.202200081 35304978

[B98] Omidian H. Wilson R. Dey Chowdhury S. (2024). Injectable biomimetic gels for biomedical applications. Biomimetics 9 (7), 418. 10.3390/biomimetics9070418 39056859 PMC11274625

[B99] O’Sullivan L. Murphy B. McLoughlin P. Duggan P. Lawlor P. G. Hughes H. (2010). Prebiotics from marine macroalgae for human and animal Health applications. Mar. Drugs 8 (7), 2038–2064. 10.3390/md8072038 20714423 PMC2920542

[B100] Pacelli S. Di Muzio L. Paolicelli P. Fortunati V. Petralito S. Trilli J. (2021). Dextran-polyethylene glycol cryogels as spongy scaffolds for drug delivery. Int. J. Biol. Macromol. 166, 1292–1300. 10.1016/j.ijbiomac.2020.10.273 33161086

[B101] Pahwa R. Goyal A. Jialal I. (2025). “Chronic inflammation”, in StatPearls [Internet]. Treasure Island, FL: StatPearls Publishing. Available online at: https://www.ncbi.nlm.nih.gov/books/NBK493173/. 29630225

[B102] Pan G. Sun S. Zhang W. Zhao R. Cui W. He F. (2016). Biomimetic design of mussel-derived bioactive peptides for dual-functionalization of titanium-based biomaterials. J. Am. Chem. Soc. 138 (45), 15078–15086. 10.1021/jacs.6b09770 27778505

[B103] Pan Q. Zong Z. Li H. Xie L. Zhu H. Wu D. (2025). Hydrogel design and applications for periodontitis therapy: a review. Int. J. Biol. Macromol. 284, 137893. 10.1016/j.ijbiomac.2024.137893 39571840

[B104] Pan X. Zong Q. Liu C. Wu H. Fu B. Wang Y. (2024). Konjac glucomannan exerts regulatory effects on macrophages and its applications in biomedical engineering. Carbohydr. Polym. 345, 122571. 10.1016/j.carbpol.2024.122571 39227106

[B105] Peng S. Niu S. Gao Q. Song R. Wang Z. Luo Z. (2024). Hydroxypropyl chitosan/ε-poly-l-lysine based injectable and self-healing hydrogels with antimicrobial and hemostatic activity for wound repair. Carbohydr. Polym. 337, 122135. 10.1016/j.carbpol.2024.122135 38710549

[B106] Picchioni F. Muljana H. (2018). Hydrogels based on dynamic covalent and non covalent bonds: a chemistry perspective. Gels 4 (1), 21. 10.3390/gels4010021 30674797 PMC6318606

[B107] Proksch J. Dal Colle M. C. S. Heinz F. Schmidt R. F. Gottwald J. Delbianco M. (2024). Impact of glycan nature on structure and viscoelastic properties of glycopeptide hydrogels. J. Pept. Sci. 30 (8), e3599. 10.1002/psc.3599 38567550

[B108] Qi J. Yan Y. Cheng B. Deng L. Shao Z. Sun Z. (2018). Enzymatic Formation of an injectable hydrogel from a glycopeptide as a biomimetic scaffold for vascularization. ACS Appl. Mater Interfaces 10 (7), 6180–6189. 10.1021/acsami.7b18535 29380599

[B109] Qi X. Wei W. Shen J. Dong W. (2019). Salecan polysaccharide-based hydrogels and their applications: a review. J. Mater. Chem. B 7 (16), 2577–2587. 10.1039/c8tb03312a 32254990

[B110] Raspa A. Gelain F. (2021). Mimicking extracellular matrix via engineered nanostructured biomaterials for neural repair. Curr. Neuropharmacol. 19 (12), 2110–2124. 10.2174/1570159x18666201111111102 33176654 PMC9185766

[B111] Rebello A. S. Mazumder N. (2024). “Chapter 8 - types of microbial polysaccharides and their characterization,” in Advanced biophysical techniques for polysaccharides characterization. Editor Mazumder N. (Academic Press), 189–219.

[B112] Rodrigues M. Kosaric N. Bonham C. A. Gurtner G. C. (2019). Wound healing: a cellular perspective. Physiol. Rev. 99 (1), 665–706. 10.1152/physrev.00067.2017 30475656 PMC6442927

[B113] Rosales A. M. Anseth K. S. (2016). The design of reversible hydrogels to capture extracellular matrix dynamics. Nat. Rev. Mater 1 (2), 15012. 10.1038/natrevmats.2015.12 29214058 PMC5714327

[B114] Sahajpal K. Shekhar S. Kumar A. Sharma B. Meena M. K. Bhagi A. K. (2022). Dynamic protein and polypeptide hydrogels based on Schiff base co-assembly for biomedicine. J. Mater Chem. B 10 (17), 3173–3198. 10.1039/d2tb00077f 35352081

[B115] Sakamoto Y. Suehiro F. Akiba I. Nishimura T. (2022). Supramolecular shear-thinning glycopeptide hydrogels for injectable enzyme prodrug therapy applications. Langmuir. 38 (18), 5883–5890. 10.1021/acs.langmuir.2c00504 35471982

[B116] Sellimi S. Maalej H. Rekik D. M. Benslima A. Ksouda G. Hamdi M. (2018). Antioxidant, antibacterial and *in vivo* wound healing properties of laminaran purified from Cystoseira barbata seaweed. Int. J. Biol. Macromol. 119, 633–644. 10.1016/j.ijbiomac.2018.07.171 30063934

[B117] Shariatinia Z. Jalali A. M. (2018). Chitosan-based hydrogels: preparation, properties and applications. Int. J. Biol. Macromol. 115, 194–220. 10.1016/j.ijbiomac.2018.04.034 29660456

[B118] Shen H. Zhang C. Meng Y. Qiao Y. Ma Y. Chen J. (2024). Biomimetic hydrogel containing copper sulfide nanoparticles and deferoxamine for photothermal therapy of infected diabetic wounds. Adv. Healthc. Mater. 13 (8), 2303000. 10.1002/adhm.202303000 38063809

[B119] Shi X. Wang K. Gao S. Zhang D. Lai C. Jin C. (2023). Facile strategy to *in situ* synthesize gallic acid-modified lignin and its utilization for fabricating conductive and self-adhesive hydrogels as strain sensors. Ind. Eng. Chem. Res. 62 (43), 17765–17775. 10.1021/acs.iecr.3c02652

[B120] Shima S. Matsuoka H. Iwamoto T. Sakai H. (1984). Antimicrobial action of.EPSILON.-poly-L-lysine. J. antibiotics 37 (11), 1449–1455. 10.7164/antibiotics.37.1449 6392269

[B121] Small R. (1989). Diclofenac sodium. Clin. Pharm. 8 (8), 545–558. 2670397

[B122] Soeiro V. Melo K. Alves M. Medeiros M. Grilo M. Almeida-Lima J. (2016). Dextran: influence of molecular weight in antioxidant properties and immunomodulatory potential. IJMS 17 (8), 1340. 10.3390/ijms17081340 27548151 PMC5000737

[B123] Stubelius A. Lee S. Almutairi A. (2019). The chemistry of boronic acids in nanomaterials for drug delivery. Acc. Chem. Res. 52 (11), 3108–3119. 10.1021/acs.accounts.9b00292 31599160

[B124] Sun H. Hong Y. Xi Y. Zou Y. Gao J. Du J. (2018). Synthesis, self-assembly, and biomedical applications of antimicrobial peptide–polymer conjugates. Biomacromolecules 19 (6), 1701–1720. 10.1021/acs.biomac.8b00208 29539262

[B125] Sun Z. Luan X. Sun Z. Li D. Hu H. Xue Q. (2024). Bioactive peptide hydrogel scaffold with high fluidity, thermosensitivity, and neurotropism in 3D spatial structure for promoted repair of spinal cord injury. Small 21, 2406990. 10.1002/smll.202406990 39513226

[B126] Teng L. Shao Z. Bai Q. Zhang X. He Y. Lu J. (2021). Biomimetic glycopolypeptide hydrogels with tunable adhesion and microporous structure for fast hemostasis and highly efficient wound healing. Adv. Funct. Mater. 31 (43), 2105628. 10.1002/adfm.202105628

[B127] Teng L. Song Y. Hu Y. Lu J. Dong C. M. (2024). Biomimetic and wound microenvironment-modulating PEGylated glycopolypeptide hydrogels for arterial massive hemorrhage and wound prohealing. Biomacromolecules 25 (7), 4317–4328. 10.1021/acs.biomac.4c00389 38829675

[B128] Teng Y. Chi J. Huang J. Li Z. Li S. Wu X. (2025). Hydrogel toughening resets biomedical application boundaries. Prog. Polym. Sci. 161, 101929. 10.1016/j.progpolymsci.2025.101929

[B129] Terriac L. Helesbeux J. J. Maugars Y. Guicheux J. Tibbitt M. W. Delplace V. (2024). Boronate ester hydrogels for biomedical applications: challenges and opportunities. Chem. Mater. 36 (14), 6674–6695. 10.1021/acs.chemmater.4c00507 39070669 PMC11270748

[B130] Thalji M. R. Ibrahim A. A. Chong K. F. Soldatov A. V. Ali G. A. M. (2022). Glycopolymer-based materials: synthesis, properties, and biosensing applications. Top. Curr. Chem. 380 (5), 45. 10.1007/s41061-022-00395-5 PMC9366760 35951265

[B131] Treenate P. Monvisade P. (2017). *In vitro* drug release profiles of pH-sensitive hydroxyethylacryl chitosan/sodium alginate hydrogels using paracetamol as a soluble model drug. Int. J. Biol. Macromol. 99, 71–78. 10.1016/j.ijbiomac.2017.02.061 28219689

[B132] Tümen Erden-Ceyda Ekentok ATICI.-B. C. S. (2021). Preparation and *in vitro* characterization of laminarin based hydrogels. jrp 25 (2), 164–172. 10.29228/jrp.7

[B133] Varaprasad K. Jayaramudu T. Kanikireddy V. Toro C. Sadiku E. R. (2020). Alginate-based composite materials for wound dressing application: a mini review. Carbohydr. Polym. 236, 116025. 10.1016/j.carbpol.2020.116025 32172843

[B134] Villarreal-Leal R. A. Healey G. D. Corradetti B. (2021). Biomimetic immunomodulation strategies for effective tissue repair and restoration. Adv. Drug Deliv. Rev. 179, 113913. 10.1016/j.addr.2021.113913 34371087

[B135] Waite J. H. Andersen N. H. Jewhurst S. Sun C. (2005). Mussel adhesion: finding the tricks worth mimicking. J. Adhesion 81 (3–4), 297–317. 10.1080/00218460590944602

[B136] Wang C. Liu Y. Fan Y. Li X. (2017). The use of bioactive peptides to modify materials for bone tissue repair. Regen. Biomater. 4 (3), 191–206. 10.1093/rb/rbx011 28596916 PMC5458541

[B137] Wang H. Heilshorn S. C. (2015). Adaptable hydrogel networks with reversible linkages for tissue engineering. Adv. Mater. 27 (25), 3717–3736. 10.1002/adma.201501558 25989348 PMC4528979

[B138] Wang H. Mu G. Cai X. Zhang X. Mao R. Jia H. (2024d). Glucopeptide superstructure hydrogel promotes surgical wound healing following neoadjuvant radiotherapy by producing NO and anticellular senescence. Adv. Healthc. Mater. 13 (20), 2400406. 10.1002/adhm.202400406 38683036

[B139] Wang J. Liu X. Wang Y. Zhang Y. Gao R. Gao Y. (2024a). Pro-regenerative glycopeptide hydrogel activates type 2 immune response for wound healing via macrophage-T cell crosstalk. Adv. Funct. Mater 34 (16). 10.1002/adfm.202307711

[B140] Wang P. Pu Y. He B. (2025a). Natural polysaccharide-based hydrogels for hemostasis and wound healing: a review. Precis. Med. Eng. 2, 100016. 10.1016/j.preme.2025.100016

[B141] Wang P. Yang Y. Yin Y. Zhang H. Shi T. Zhang W. (2025b). Thermoresponsive injectable microsphere glycopeptide hydrogels for remodeling dynamic cell microenvironments. ACS Appl. Polym. Mater 7 (3), 1236–1248. 10.1021/acsapm.4c02740

[B142] Wang Q. Li M. Lu Q. Tao R. Liao J. Zhao J. (2024b). *Lycium barbarum-* derived polysaccharides alleviate DNA damage and oxidative stress caused by ultraviolet radiation in corneal epithelial cells. Curr. Eye Res. 49 (11), 1123–1130. 10.1080/02713683.2024.2366309 39444111

[B143] Wang R. Xu D. lei Liang L. Xu T. ting Liu W. Ouyang P. kai (2016). Enzymatically crosslinked epsilon-poly-L-lysine hydrogels with inherent antibacterial properties for wound infection prevention. Rsc Adv. 6 (11), 8620–8627. 10.1039/c5ra15616e

[B144] Wang X. Wei Z. Wu Z. Li Y. Miao C. Cao Z. (2024c). Thermosensitive injectable dual drug-loaded chitosan-based hydrogels for treating bacterial endometritis. ACS Biomater. Sci. Eng. 10 (12), 7516–7526. 10.1021/acsbiomaterials.4c01729 39545662

[B145] Wang Y. Wang J. Gao R. Liu X. Feng Z. Zhang C. (2022). Biomimetic glycopeptide hydrogel coated PCL/nHA scaffold for enhanced cranial bone regeneration via macrophage M2 polarization-induced osteo-immunomodulation. Biomaterials 285, 121538. 10.1016/j.biomaterials.2022.121538 35504180

[B146] Wang Y. Zeng K. (2019). Natural products as a crucial source of anti-inflammatory drugs: recent trends and advancements. Tradit. Med. Res. 4 (5), 257–268–268. 10.53388/tmr20190831133

[B147] Wang Y. Zhao Y. He J. Sun C. Lu W. Zhang Y. (2023). Doubling growth of egg-box structure during Calcium-mediated molecular assembly of alginate. J. Colloid Interface Sci. 634, 747–756. 10.1016/j.jcis.2022.12.096 36563431

[B148] Wang Z. Deng X. Ding J. Zhou W. Zheng X. Tang G. (2018). Mechanisms of drug release in pH-sensitive micelles for tumour targeted drug delivery system: a review. Int. J. Pharm. 535 (1–2), 253–260. 10.1016/j.ijpharm.2017.11.003 29113804

[B149] Wu Y. Wang Y. Long L. Hu C. Kong Q. Wang Y. (2022). A spatiotemporal release platform based on pH/ROS stimuli-responsive hydrogel in wound repairing. J. Control Release 341, 147–165. 10.1016/j.jconrel.2021.11.027 34813880

[B150] Wu Y. Wang Y. Zheng C. Hu C. Yang L. Kong Q. (2023). A versatile glycopeptide hydrogel promotes chronic refractory wound healing through bacterial elimination, sustained oxygenation, immunoregulation, and neovascularization. Adv. Funct. Mater 33 (49). 10.1002/adfm.202305992

[B151] Wu Y. Zhao S. Wang J. Chen Y. Li H. Li J. ping (2024). Methods for determining the structure and physicochemical properties of hyaluronic acid and its derivatives: a review. Int. J. Biol. Macromol. 282, 137603. 10.1016/j.ijbiomac.2024.137603 39542327

[B152] Xia H. Dong Z. Tang Q. Ding R. Bai Y. Zhou K. (2023). Glycopeptide‐based multifunctional hydrogels promote diabetic wound healing through pH regulation of microenvironment. Adv. Funct. Mater. 33 (29), 2215116. 10.1002/adfm.202215116

[B153] Xie Y. Zhao J. Huang R. Qi W. Wang Y. Su R. (2016). Calcium-ion-triggered Co-assembly of peptide and polysaccharide into a hybrid hydrogel for drug delivery. Nanoscale Res. Lett. 11 (1), 184. 10.1186/s11671-016-1415-8 27067732 PMC4828348

[B154] Xiong Y. Mi B. B. Lin Z. Hu Y. Q. Yu L. Zha K. K. (2022). The role of the immune microenvironment in bone, cartilage, and soft tissue regeneration: from mechanism to therapeutic opportunity. Mil. Med. Res. 9 (1), 65. 10.1186/s40779-022-00426-8 36401295 PMC9675067

[B155] Xiu A. Kong Y. Zhou M. Zhu B. Wang S. Zhang J. (2010). The chemical and digestive properties of a soluble glucan from Agrobacterium sp. ZX09. Carbohydr. Polym. 82 (3), 623–628. 10.1016/j.carbpol.2010.05.027

[B156] Xu N. Gao Y. Li Z. Chen Y. Liu M. Jia J. (2023). Immunoregulatory hydrogel decorated with Tannic acid/Ferric ion accelerates diabetic wound healing via regulating Macrophage polarization. Chem. Eng. J. 466, 143173. 10.1016/j.cej.2023.143173

[B157] Xu X. D. Liang L. Cheng H. Wang X. H. Jiang F. G. Zhuo R. X. (2012). Construction of therapeutic glycopeptide hydrogel as a new substitute for antiproliferative drugs to inhibit postoperative scarring formation. J. Mater Chem. 22 (35), 18164–18171. 10.1039/c2jm32519e

[B158] Yan W. Dai W. Cheng J. Fan Y. Wu T. Zhao F. (2021b). Advances in the mechanisms affecting meniscal avascular zone repair and therapies. Front. Cell Dev. Biol. 9, 758217. 10.3389/fcell.2021.758217 34778268 PMC8581462

[B159] Yan Y. Li Y. Zhang Z. Wang X. Niu Y. Zhang S. (2021a). Advances of peptides for antibacterial applications. Colloids Surfaces B Biointerfaces 202, 111682. 10.1016/j.colsurfb.2021.111682 33714188

[B160] Yang R. Huang J. Zhang W. Xue W. Jiang Y. Li S. (2021a). Mechanoadaptive injectable hydrogel based on poly(γ-glutamic acid) and hyaluronic acid regulates fibroblast migration for wound healing. Carbohydr. Polym. 273, 118607. 10.1016/j.carbpol.2021.118607 34561006

[B161] Yang R. Liu X. Ren Y. Xue W. Liu S. Wang P. (2021b). Injectable adaptive self-healing hyaluronic acid/poly (γ-glutamic acid) hydrogel for cutaneous wound healing. Acta Biomater. 127, 102–115. 10.1016/j.actbio.2021.03.057 33813093

[B162] Yao X. Hu Y. Lin M. Peng K. Wang P. Gao Y. (2023). Self-assembling peptide RADA16: a promising scaffold for tissue engineering and regenerative medicine. Nanomedicine 18 (19), 1305–1326. 10.2217/nnm-2023-0161 37750388

[B163] Yao Y. Zaw A. M. Anderson D. E. Hinds M. T. Yim E. K. (2020). Fucoidan functionalization on poly (vinyl alcohol) hydrogels for improved endothelialization and hemocompatibility. Biomaterials 249, 120011. 10.1016/j.biomaterials.2020.120011 32304872 PMC7748769

[B164] Ye K. Y. Black L. D. (2011). Strategies for tissue engineering cardiac constructs to affect functional repair following myocardial infarction. J Cardiovasc Trans Res 4 (5), 575. 10.1007/s12265-011-9303-1 PMC3182851 21818697

[B165] Yokoi H. Kinoshita T. Zhang S. (2005). Dynamic reassembly of peptide RADA16 nanofiber scaffold. Proc. Natl. Acad. Sci. 102 (24), 8414–8419. 10.1073/pnas.0407843102 15939888 PMC1150805

[B166] Zhang C. Wu B. Zhou Y. Zhou F. Liu W. Wang Z. (2020b). Mussel-inspired hydrogels: from design principles to promising applications. Chem. Soc. Rev. 49 (11), 3605–3637. 10.1039/c9cs00849g 32393930

[B167] Zhang K. Feng Q. Fang Z. Gu L. Bian L. (2021). Structurally dynamic hydrogels for biomedical applications: pursuing a fine balance between macroscopic stability and microscopic dynamics. Chem. Rev. 121 (18), 11149–11193. 10.1021/acs.chemrev.1c00071 34189903

[B168] Zhang W. Shao Q. Zhong H. Yang Y. Li R. Liu Y. (2025). Glycopeptide microneedles triggering the ECM process to promote fibroblast viability for anti-aging treatments. Biomater. Adv. 168, 214124. 10.1016/j.bioadv.2024.214124 39616682

[B169] Zhang W. Wang R. Sun Z. Zhu X. Zhao Q. Zhang T. (2020a). Catechol-functionalized hydrogels: biomimetic design, adhesion mechanism, and biomedical applications. Chem. Soc. Rev. 49 (2), 433–464. 10.1039/c9cs00285e 31939475 PMC7208057

[B170] Zhang X. Wu Y. Gong H. Xiong Y. Chen Y. Li L. (2024). A multifunctional herb-derived glycopeptide hydrogel for chronic wound healing. Small 30, 2400516. 10.1002/smll.202400516 38686688

[B171] Zhao X. Kwan J. Y. Y. Yip K. Liu P. P. Liu F. F. (2020). Targeting metabolic dysregulation for fibrosis therapy. Nat. Rev. Drug Discov. 19 (1), 57–75. 10.1038/s41573-019-0040-5 31548636

[B172] Zhao Y. Jalili S. (2022). Dextran, as a biological macromolecule for the development of bioactive wound dressing materials: a review of recent progress and future perspectives. Int. J. Biol. Macromol. 207, 666–682. 10.1016/j.ijbiomac.2022.02.114 35218804

[B173] Zhao Y. Song S. Ren X. Zhang J. Lin Q. Zhao Y. (2022). Supramolecular adhesive hydrogels for tissue engineering applications. Chem. Rev. 122 (6), 5604–5640. 10.1021/acs.chemrev.1c00815 35023737

[B174] Zhao Y. Yokoi H. Tanaka M. Kinoshita T. Tan T. (2008). Self-assembled pH-responsive hydrogels composed of the RATEA16 peptide. Biomacromolecules 9 (6), 1511–1518. 10.1021/bm701143g 18498190

[B175] Zhao Z. Feng M. Wan J. Zheng X. Teng C. Xie X. (2021). Research progress of epigallocatechin-3-gallate (EGCG) on anti-pathogenic microbes and immune regulation activities. Food Funct. 12 (20), 9607–9619. 10.1039/d1fo01352a 34549212

[B176] Zhao Z. Wu C. Huangfu Y. Zhang Y. Zhang J. Huang P. (2024b). Bioinspired glycopeptide hydrogel reestablishing bone homeostasis through mediating osteoclasts and osteogenesis in periodontitis treatment. ACS Nano 18 (43), 29507–29521. 10.1021/acsnano.4c05677 39401162

[B177] Zhao Z. Xia X. Liu J. Hou M. Liu Y. Zhou Z. (2024a). Cartilage-inspired self-assembly glycopeptide hydrogels for cartilage regeneration via ROS scavenging. Bioact. Mater 32, 319–332. 10.1016/j.bioactmat.2023.10.013 37869724 PMC10589380

[B178] Zhou W. Duan Z. Zhao J. Fu R. Zhu C. Fan D. (2022). Glucose and MMP-9 dual-responsive hydrogel with temperature sensitive self-adaptive shape and controlled drug release accelerates diabetic wound healing. Bioact. Mater. 17, 1–17. 10.1016/j.bioactmat.2022.01.004 35386439 PMC8958327

[B179] Zhou Y. X. Gong X. H. Zhang H. Peng C. (2020). A review on the pharmacokinetics of paeoniflorin and its anti-inflammatory and immunomodulatory effects. Biomed. and Pharmacother. 130, 110505. 10.1016/j.biopha.2020.110505 32682112

[B180] Zhu H. Liu R. Shang Y. Sun L. (2023). Polylysine complexes and their biomedical applications. Eng. Regen. 4 (1), 20–27. 10.1016/j.engreg.2022.11.001

[B181] Zhu Y. Liu L. Sun Z. Ji Y. Wang D. Mei L. (2021). Fucoidan as a marine-origin prebiotic modulates the growth and antibacterial ability of Lactobacillus rhamnosus. Int. J. Biol. Macromol. 180, 599–607. 10.1016/j.ijbiomac.2021.03.065 33757852

[B182] Zhu Z. Ye H. Zhang K. He G. Pan Z. Xian Y. (2024). Naturally derived injectable dual-cross-linked adhesive hydrogel for acute hemorrhage control and wound healing. Biomacromolecules 25 (4), 2574–2586. 10.1021/acs.biomac.4c00105 38525818

